# Hyperactive nanobacteria with host-dependent traits pervade Omnitrophota

**DOI:** 10.1038/s41564-022-01319-1

**Published:** 2023-03-16

**Authors:** Cale O. Seymour, Marike Palmer, Eric D. Becraft, Ramunas Stepanauskas, Ariel D. Friel, Frederik Schulz, Tanja Woyke, Emiley Eloe-Fadrosh, Dengxun Lai, Jian-Yu Jiao, Zheng-Shuang Hua, Lan Liu, Zheng-Han Lian, Wen-Jun Li, Maria Chuvochina, Brianna K. Finley, Benjamin J. Koch, Egbert Schwartz, Paul Dijkstra, Duane P. Moser, Bruce A. Hungate, Brian P. Hedlund

**Affiliations:** 1 School of Life Sciences, University of Nevada, Las Vegas, Las Vegas, NV USA; 2 Bigelow Laboratory for Ocean Sciences, East Boothbay, ME USA; 3 Department of Biology, University of North Alabama, Florence, AL USA; 4 DOE Joint Genome Institute, Berkeley, CA USA; 5 Lawrence Berkeley National Laboratory, Berkeley, CA USA; 6 State Key Laboratory of Biocontrol, Guangdong Provincial Key Laboratory of Plant Resources and Southern Marine Science and Engineering Guangdong Laboratory (Zhuhai), School of Life Sciences, Sun Yat-Sen University, Guangzhou, People’s Republic of China; 7 Department of Environmental Science and Engineering, University of Science and Technology of China, Hefei, People’s Republic of China; 8 State Key Laboratory of Desert and Oasis Ecology, Xinjiang Institute of Ecology and Geography, Chinese Academy of Sciences, Urumqi, People’s Republic of China; 9 Australian Centre for Ecogenomics, School of Chemistry and Molecular Biosciences, University of Queensland, Brisbane, Queensland Australia; 10 Center for Ecosystem Science and Society (ECOSS), Northern Arizona University, Flagstaff, AZ USA; 11 Division of Hydrologic Sciences, Desert Research Institute, Las Vegas, NV USA; 12 Nevada Institute of Personalized Medicine, Las Vegas, NV USA; 13 Present Address: Department of Ecology and Evolutionary Biology, University of California, Irvine, CA USA

**Keywords:** Microbial ecology, Microbiology

## Abstract

Candidate bacterial phylum Omnitrophota has not been isolated and is poorly understood. We analysed 72 newly sequenced and 349 existing Omnitrophota genomes representing 6 classes and 276 species, along with Earth Microbiome Project data to evaluate habitat, metabolic traits and lifestyles. We applied fluorescence-activated cell sorting and differential size filtration, and showed that most Omnitrophota are ultra-small (~0.2 μm) cells that are found in water, sediments and soils. Omnitrophota genomes in 6 classes are reduced, but maintain major biosynthetic and energy conservation pathways, including acetogenesis (with or without the Wood-Ljungdahl pathway) and diverse respirations. At least 64% of Omnitrophota genomes encode gene clusters typical of bacterial symbionts, suggesting host-associated lifestyles. We repurposed quantitative stable-isotope probing data from soils dominated by andesite, basalt or granite weathering and identified 3 families with high isotope uptake consistent with obligate bacterial predators. We propose that most Omnitrophota inhabit various ecosystems as predators or parasites.

## Abstract (web summary)

A compendium of Omnitrophota genomes is coupled with cell size and qSIP analyses to predict the physiology and lifestyle of this enigmatic phylum.

## Main

The candidate bacterial phylum Omnitrophota (synonyms: OP3, Omnitrophica, Omnitrophicaeota; herein formally named Omnitrophota) has been identified in 16S ribosomal RNA gene surveys^1,2^, particularly in water and sediment. Published 16S rRNA gene^2,3^- and metagenome-based^2,4^ studies have placed Omnitrophota in the Planctomycetota–Verrucomicrobiota–Chlamydiota (PVC) superphylum. Omnitrophota have not been isolated and only two species have been microscopically observed. ‘*Candidatus* Omnitrophus magneticus’ SKK-01 (ref. ^5^) was described as a large, ovoid putatively free-living magnetic bacterium containing sulfur inclusions (herein referred to as SKK-01). ‘*Ca.* Velamenicoccus archaeovorus’ LiM^6^ was identified as a small (0.2–0.3 μm) coccus present in methanogenic, limonene-degrading enrichment cultures both as free cells and as an epibiont of other bacteria or archaea, including *Methanosaeta*^6^. When living as an epibiont, *V. archaeovorus* has a different transcriptional output, increases ribosome content and seems to damage or kill host cells^6^. Given the different lifestyles of SKK-01 and *V. archaeovorus* LiM, it is unclear whether either is a meaningful representative for the phylum.

Previous reports have proposed that metabolic capabilities of Omnitrophota single-amplified genomes (SAGs) and metagenome-assembled genomes (MAGs) include heterotrophic aerobic respiration or acetogenesis^4–8^. Comparative genomics of 14 Omnitrophota MAGs from an Antarctic lake and a single MAG from Black Sea sediments suggested that all were obligate fermenters^7,9^. So far, however, a systematic effort to interpret genomics data within the context of the phylum Omnitrophota has been lacking.

Here we analyse a compendium of SAGs (75) and MAGs (346), together with cell size and in situ metabolism studies to present a more comprehensive picture of Omnitrophota biology.

## Taxonomic distribution of Omnitrophota

**Fig. 1:**
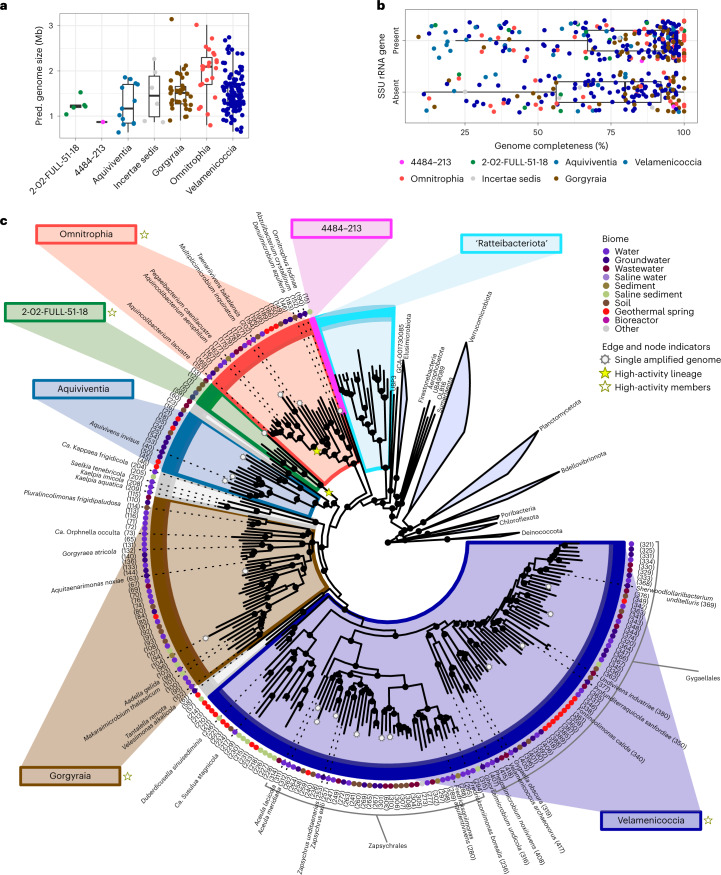
Omnitrophota genomes and taxonomy. **a**, Genome size estimates for ≥90% complete Omnitrophota genomes. **b**, Genome completeness and 16S rRNA gene detection statistics of all Omnitrophota genomes included in this analysis. Colours represent classes. In **a** and **b**, boxplots represent interquartile ranges, horizontal/vertical bars are means and vertical/horizontal bars are 95% confidence intervals. **c**, Maximum-likelihood phylogeny constructed from the concatenated Bac120 marker set of 204 Omnitrophota species representatives. The number within parentheses at the end of each tip corresponds to the genome ID in Supplementary Table 1. Dotted nodes indicate SH-aLRT support ≥80% and UFboot support ≥95%. Source data

Genomes (421) classified as Omnitrophota were gathered from our own new data (72 genomes) and existing data (349 genomes), including 75 SAGs and 346 MAGs (Fig. 1 and Supplementary Table 1). The genomes originated from environmental biome samples, including lake or river water (111), groundwater (97), geothermal sediments (64), bulk soils (59), wastewater (37) and marine or otherwise saline sediments (30).

**Extended Data Fig. 1:**
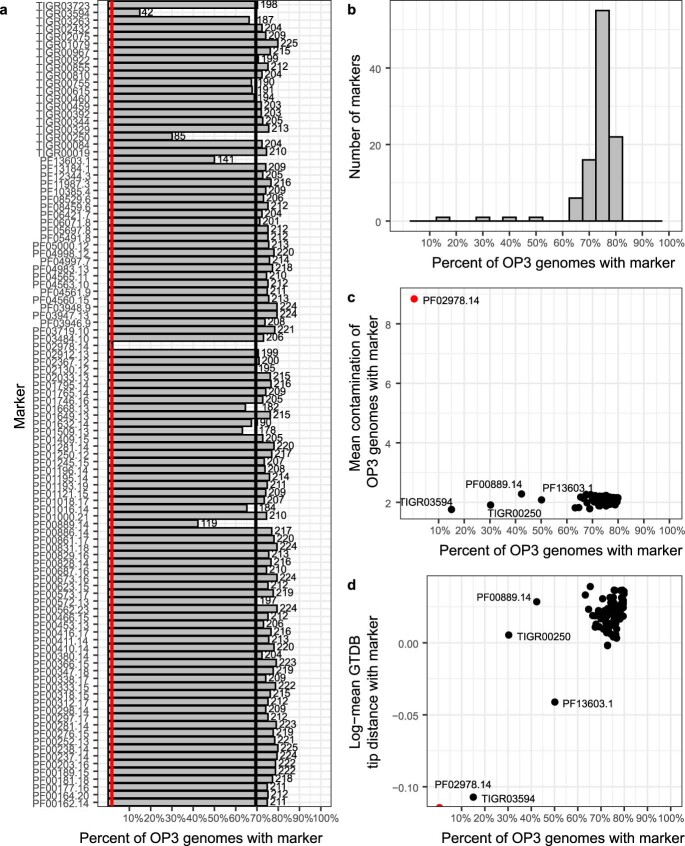
General bacterial marker set analysis. CheckM General bacterial marker set analysis. (a) Barplot showing the percent of Omnitrophota genomes with a given marker gene. The red line indicates the mean (uncorrected) contamination of all Omnitrophota genomes. The black line indicates the mean (uncorrected) completeness estimate of all Omnitrophota genomes. The number at the top of the bar indicates the number of genomes that encode a given marker. (b) Histogram showing the distribution of marker genes, according to how many of the Omnitrophota genomes encoded them. (c) Dotplot showing the percent of Omnitrophota genomes with a given marker compared to the mean contamination of all Omnitrophota genomes encoding that marker. (d) Dotplot comparing the percent of genomes that encode a given marker and the mean distance on the GTDB tree between those genomes. Source data

**Extended Data Fig. 2:**
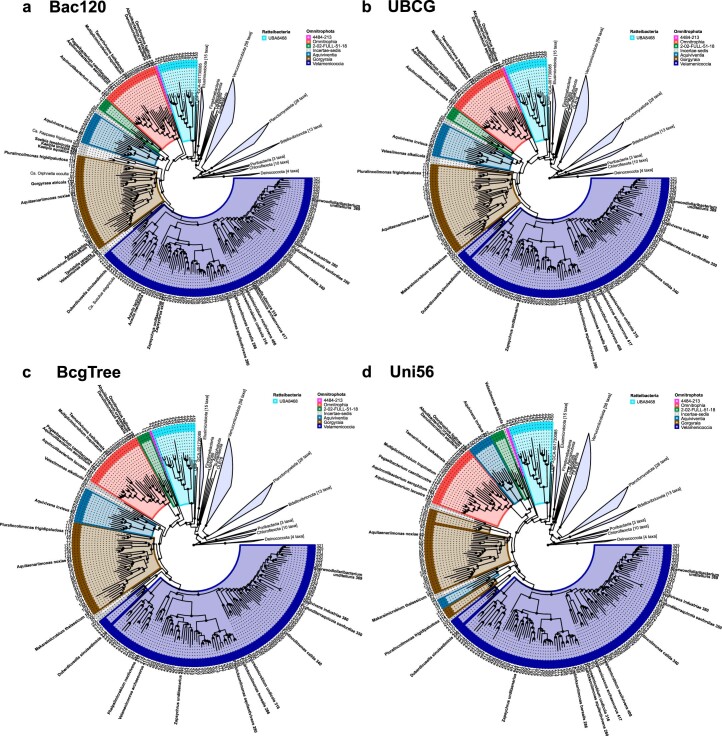
Concatenated protein phylogenies with class-level annotations. Phylogenetic trees constructed from the Bac120, UBCG, BcgTree and Uni156 marker sets, painted with Class-level taxonomy. Tips reflect genome identifiers used in the study along with proposed taxonomic names in bold, corresponding to Supplementary Table 1. Supported nodes (SH-aLRT ≥ 80% and UFboot ≥ 95%) are indicated with a black dot.

Our compendium of Omnitrophota genomes is diverse. Using a 95% average nucleotide identity (ANI) threshold^10^, we identified 276 putative species clusters, with 204 of these including at least one high- or medium-quality^11^ genome, as assessed using CheckM2 or a customized CheckM marker set (Fig. 1b, Extended Data Fig. 1 and Supplementary Fig. 1). The highest-quality member of each species cluster was selected for phylogenetic analysis using three ‘bacteria-specific’ marker sets (Bac120 (ref. ^12^), UBCG^13^, bcgTree (ref. ^14^)) and the ‘universal’ marker set Uni156 (ref. ^15^) (Extended Data Fig. 2 and Supplementary Figs. 1–3). Concordance among bacteria-specific marker trees confirmed the Omnitrophota as members of the PVC superphylum, rejected ‘Ratteibacteriota’ as Omnitrophota (Supplementary Note 1) and enabled revision of the Genome Taxonomy Database (GTDB) taxonomy^12,16^ to eliminate poly- and paraphyletic taxa across all three conserved marker gene trees (Supplementary Fig. 4). Finally, we proposed new ranks between genus and class by evaluating relative evolutionary divergence and average amino acid identity (AAI)^17^ (Supplementary Table 3).

Our refined taxonomic classification resulted in 6 classes, 25 orders, 52 families, 146 genera and 204 species represented by a medium- or high-quality genome, which substantially expands the previously published 12 *Candidatus* genera and species in the Omnitrophota (Supplementary Tables 1–3).

To name taxa using SeqCode^18^, we developed a system of nomenclature on the basis of high-quality genomic assemblies that had near-full-length 16S rRNA and 23S rRNA genes, using the Damerau-Levenshtein distance algorithm to ensure new taxonomic names are unique (Supplementary Tables 1 and 3, and Supplementary Fig. 5). In some cases, historical data were used as nomenclatural types to retain historical names, and some previous names were rejected due to low data quality or synonymy (Online Methods). We named 36 species using this approach, and four classes*:* Velamenicoccia, Omnitrophia, Gorgyraia and Aquiviventia (Fig. 1). For the two remaining classes, no full-length 16S rRNA gene could be matched to an otherwise high-quality genome, so we conservatively retained the alphanumeric designations 2-02-FULL-51-18 and 4484-213. Nine species clusters were phylogenetically unstable and were assigned ‘incertae sedis’ status at the class level.

## Omnitrophota are present in soils and aquatic environments

**Extended Data Fig. 3:**
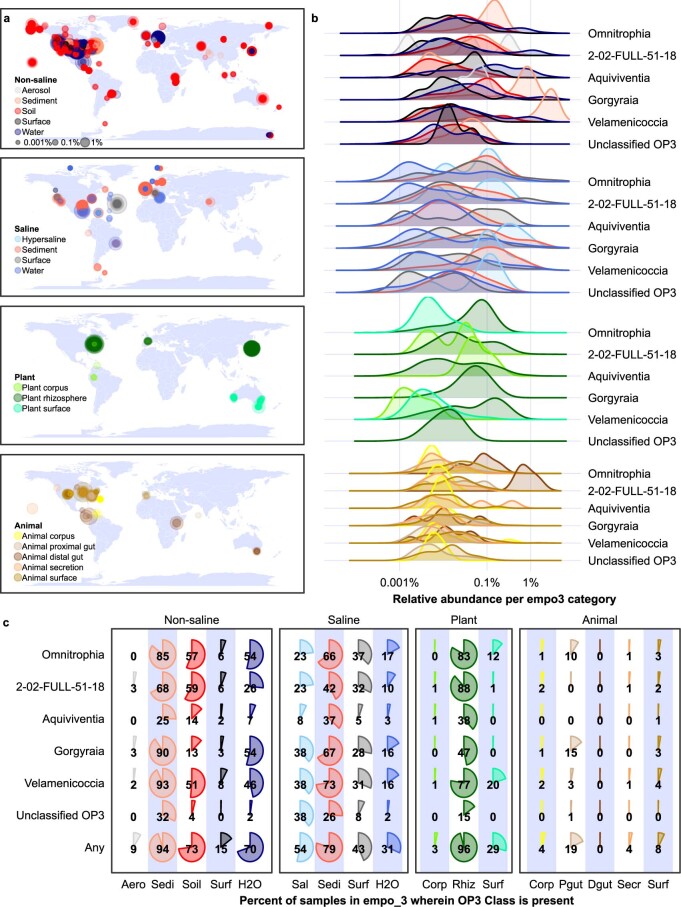
Global distribution of Omnitrophota. (a) Maps showing the coordinates of Earth Microbiome Project (EMP) samples in which Omnitrophota sequence variants were observed in (from top to bottom) EMP environmental ontology (EMPO) level-2 categories: Non-saline, Saline, Plant-associated, and Animal-associated. Bubbles depict relative abundance of the phylum and are colored to indicate EMPO level 3. (b) Log10-scale distribution of percent relative abundances of each class of Omnitrophota within each EMPO-3 category. (c) Multi-pie chart displaying the percent of EMP samples containing Omnitrophota sequences in each EMPO-3 category (x-axis). ‘Aero’ refers to aerosol samples, ‘Sedi’ sediment, ‘Surf’ surface, ‘H2O’ water, ‘Sal’ hypersaline, ‘Corp’ corpus, ‘Rhiz’ rhizosphere, ‘Pgut’ proximal gut, ‘Dgut’ distal gut, ‘Secr’ secretion. Source data

We applied a QIIME2 (ref. ^19^)-compatible naïve-Bayesian classifier for Omnitrophota (Online Methods) to 25,744 samples from the Earth Microbiome Project (EMP)^20^ and found that 65% of environmental samples contain Omnitrophota sequence variants (SV) (Extended Data Fig. 3). Omnitrophota were prevalent in non-saline environments, including waters (70% of EMP samples), sediments (94% of EMP samples) and soils (73% of EMP samples), with enrichment in rhizosphere soils (96% of EMP samples), although typically at low relative abundance (<0.1%). Omnitrophota were almost absent from animal-associated samples, and where they were abundant, samples were from the alimentary tract of sediment-consuming benthic fishes in the genus *Fundulus* (Extended Data Fig. 3).

The broad distribution of Omnitrophota in EMP biomes agrees with the provenance of the genomic assemblies. In EMP samples, Velamenicoccia and Omnitrophia are the most widely distributed classes, followed by Gorgyraia and class 2-02-FULL-51-18, with Aquiviventia being the least common. Non-saline sediments displayed the lowest taxonomic specificity, with each of the four common classes occurring in >70% of EMP samples. Soils displayed the highest taxonomic specificity, with only Omnitrophia, Velamenicoccia and class 2-02-FULL-51-18 occurring at high frequencies. Omnitrophia, Velamenicoccia and Gorgyraia occurred at higher relative abundances in anoxic aquatic environments relative to oxic waters. We propose a broad physicochemical niche for Omnitrophota, with most members being part of the rare biosphere.

## Most Omnitrophota are nanobacteria

**Fig. 2:**
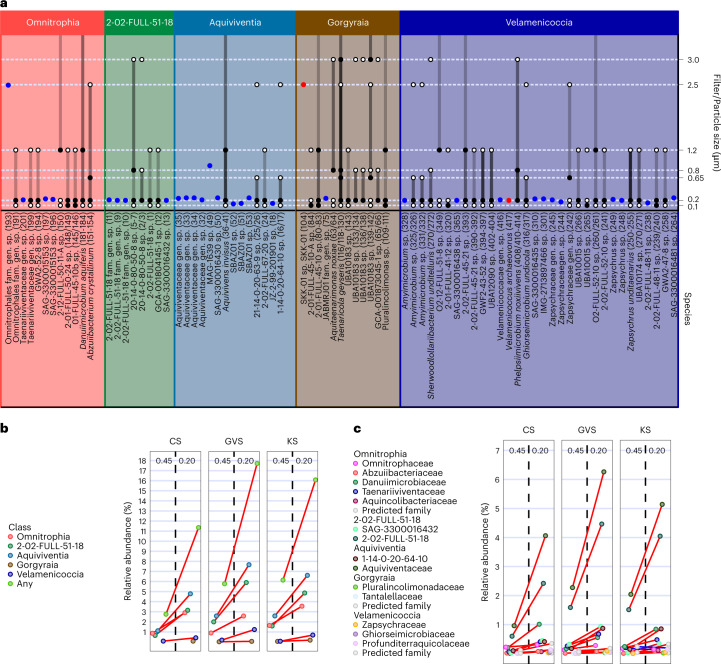
Omnitrophota cell size. **a**, Genomes associated with microscopically observed organisms are indicated with red circles. SAGs with associated particle size estimates are indicated as blue dots. Cell size estimates associated with MAGs from Rifle, Colorado^108,109^ and Crystal Geyser, Utah^110^ are based on serial-filtered samples. Filters are shown as black or white circles: filters represented by filled circles retained the organism; unfilled circles did not have Omnitrophota MAGs. Lines connecting dots represent the estimated cell size ranges given the observed data. **b**, Relative abundance of 16S rRNA genes in filtrates from Cave Spring (CS), Kiup Spring (KS) and Grapevine Spring (GVS). Red lines indicate an increase in relative abundance from the 0.45 μm filter to the 0.2 μm filter. **c**, The same calculation performed at the family level. Source data

A previous study suggested that some Omnitrophota cells are small^21^ (flow cytometry and filtration plus 16S rRNA sequencing), and analysis of enrichment cultures containing *V. archaeovorus* LiM revealed cells that were 200–300 nm in diameter^6^. Here we estimated cell diameters using fluroescence-activated cell sorting (FACS) for 36 SAGs and found that most cells are ~0.2 μm (Fig. 2a), similar to nano-sized Patescibacteria and DPANN archaea^22^. Velamenicoccia cells (12/12) and class 2-02-FULL-51-18 cells (4/4) were universally <0.3 μm, as were most FACS-sorted Aquiviventia (9/10), Omnitrophia (3/4) and Gorgyraia (1/2). Only a few cells of Aquiviventia (1/10), Omnitrophia (1/4) and Gorgyraia (1/2) were >0.3 μm. On the basis of several marker gene sets, we concluded that the SAGs were not contaminated (Extended Data Fig. 1, and Supplementary Figs. 1 and 2), suggesting that either single cells were >0.3 μm or they might be dividing or are single-species aggregates. MAGs (112) from serially filtered cells revealed some species small enough to pass through a 0.2 μm filter in the Velamenicoccia (3), Gorgyraia (3) and Omnitrophia (2) classes (Fig. 2a). Similar to the SAGs, only a few MAGs were recovered from larger size fractions (>0.65 μm) from across the phylum, including Velamenicoccia (5 MAGs), Gorgyraia (8 MAGs), Omnitrophia (3 MAGs), Aquiviventia (1 MAG) and class 2-02-FULL-51-18 (1 MAG); however, whether these represent single cells >0.65 μm or aggregates is unclear. We note that 16S rRNA gene amplicon sequencing of abundant Omnitrophota populations from serial-filtered source water from Cave Spring, Kiup Spring and Grapevine Springs, all in the Spring Mountains of Nevada, produced similar results: all five classes and 13/14 families were more abundant on 0.2 μm filters than on 0.45 μm filters in all springs following tandem filtration (Fig. 2b,c). Together, these results show that cells of all classes of Omnitrophota are frequently among the smallest known cells (Supplementary Table 4).

## Reduced genomes but relatively complete metabolisms

**Fig. 3:**
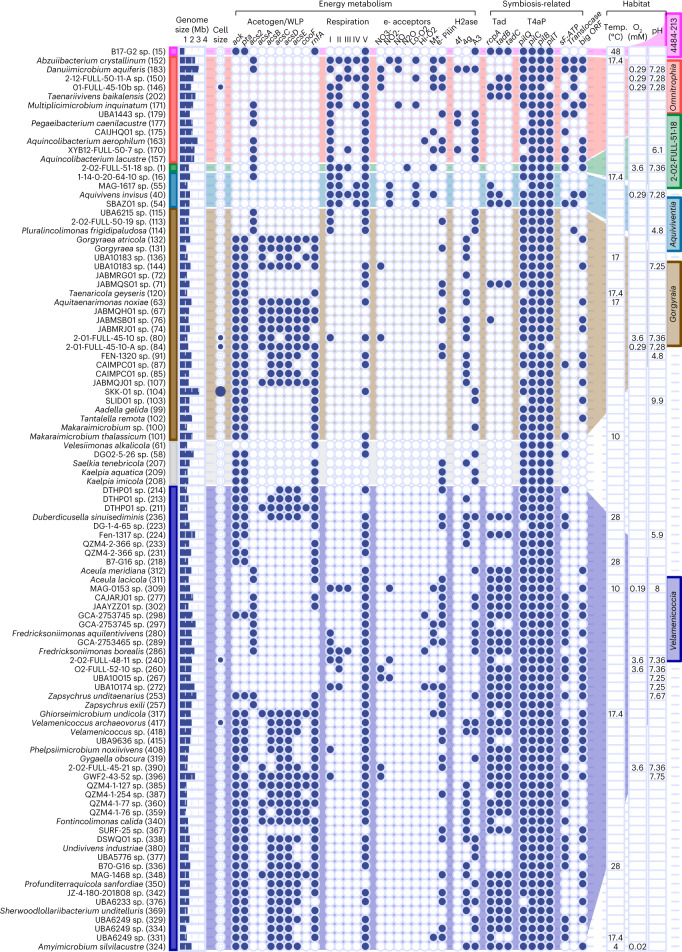
Predicted physiology and environmental data. Predicted physiology and environmental data for the highest-quality genome for each species group that has a near-complete (≥90%) genome. Bars to the right of taxon names and in background reflect classes (Fig. 1). Numbers in parentheses next to taxon names are unique genome identifiers as discussed in text. ‘Genome size’ indicates the observed size of each genome. ‘Cell size’ indicates evidence that the genome was sequenced from small cells (<0.5 μm, filled small circle) or large cells (>0.5 μm, filled large circle). ‘Acetogen/WLP’, Wood-Ljungdahl pathway and acetogenesis; ‘acs2’, acetyl-CoA synthetase; ‘acsABCDE’, CO dehydrogenase/acetyl-CoA synthase; ‘Respiration’, ‘e- acceptors’ and ‘H2ase’ (hydrogenase) indicate genes predicted to encode proteins involved in energy metabolism. ‘Lo-O2’, cytochrome c oxidase complex; ‘Hi-O2’, cytochrome bd ubiquinol; ‘M+’, metal-reducing cytochromes; ‘e- Pilin’, conductive pili. Symbiosis-related genes include ‘T4aP’ (type-4a pilus), ‘Tad’ (tight-adherence pilus), ‘sF-ATP (‘symbiotic’ type 2/3 F_o_F_1_ ATPase α-subunit), ‘Translocase’ (ATP/ADP translocase) and ‘big ORF’ (indicating the presence of a large ORF). ‘Temp’, ‘O_2_’ and ‘pH’ indicate the observed temperature, oxygen concentration (mM) and pH of the sample from which each genome was sequenced. Data for additional Omnitrophota genomes are summarized in Supplementary Fig. 10. Source data

**Extended Data Fig. 4:**
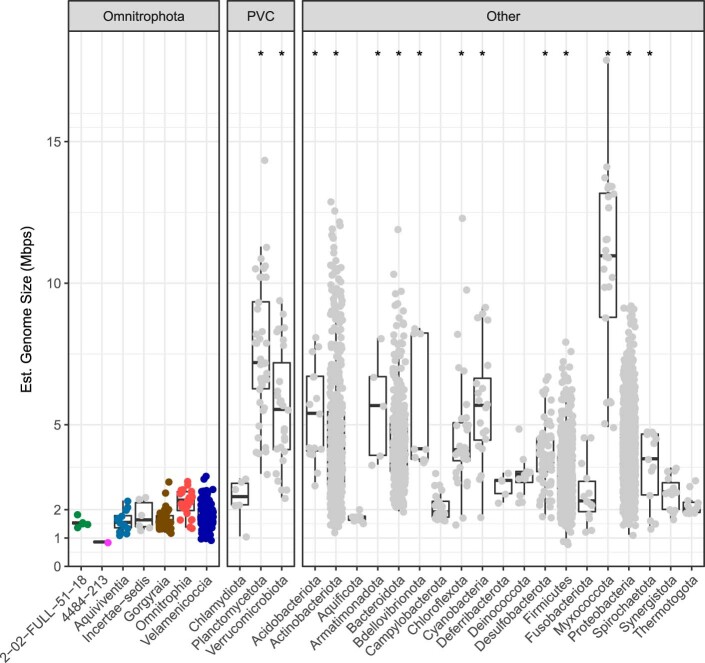
Genome sizes of Omnitrophota and other phyla. Only ≥50% complete, ≤10% contaminated genomes were used. Genome size was estimated from observed assembly size, completeness, and contamination. The centermost divider of each boxplot represents the median. Upper and lower bounds of each box represent Q3 and Q2 respectively. Whiskers extend beyond Q3 and Q2 by ±1.5 IQR. ‘*‘ indicates a significant (p < 0.05; one-way ANOVA with Tukey’s Post-Hoc test) difference in genome size between Omnitrophota and each outgroup phylum. N = 3309 (191 Omnitrophota and 3118 outgroup phyla). Source data

Small bacteria often have genomes of reduced size and function^23^. The predicted genome sizes of Omnitrophota range between 0.86 and 3.20 Mb (Figs. 1a and 3, and Extended Data Fig. 4), smaller than those of nearly all other bacterial phyla, including Planctomycetota and Verrucomicrobiota but not Chlamydiota (*P* < 0.05, analysis of variance (ANOVA) and post-hoc Tukey’s honestly significant difference (HSD). Most individual Omnitrophota genomes are in the smallest 5% of all bacterial genomes, except for those from the class Omnitrophia (mean 2.26 Mb; Fig. 1a).

Despite overall genome reduction, no Clusters of Orthologous Groups (COG) category was reduced in richness or percentage in Omnitrophota genomes compared with PVC genomes (Supplementary Fig. 6), and biosynthetic pathways were generally complete (Supplementary Table 5 and Supplementary Figs. 7–9). For example, >75% of 204 high- and medium-quality species representative genomes encoded biosynthetic pathways for nucleotides, all 20 amino acids, NAD, glutathione, pantothenate, coenzyme A, riboflavin, tetrahydrofolate and thiamine. Biosynthetic pathways for heme, cobalamin and biotin were present but not universal (that is, <75% of genomes). Biosynthetic pathways for pyridoxal-5P (M00124) were missing or incomplete across the phylum. C5-isoprenoid biosynthesis (M00096) was complete or near-complete and 4-hydroxybenzoate polyprenyltransferase was detected in >50% of genomes of the Omnitrophia and Aquiviventia, satisfying the requirements to commit 4-hydroxybenzoate to ubiquinone biosynthesis via 4-hydroxy-3-polyprenylbenzoate (M00017). However, chorismate-pyruvate lyase was not detected, so ubiquinone biosynthesis may initiate from an alternate source of 4-hydroxybenzoate rather than chorismate. Menaquinone biosynthetic pathways (M00016) were present in some Velamenicoccia genomes, particularly Zapsychrales. Quinone biosynthesis genes were absent from genomes of 2-02-FULL-51-18 species. Compared with other species from the PVC superphylum, genes not mapping to COGs were reduced in both richness and percentage (*P* < 0.05, one-way ANOVA and post-hoc Tukey’s HSD) (Supplementary Fig. 6). These analyses show that this phylum has a propensity towards small, streamlined genomes that retain most genes essential for a free-living lifestyle, including energy conservation.

## Gorgyraia and Velamenicoccia genomes encode acetogenesis

**Fig. 4:**
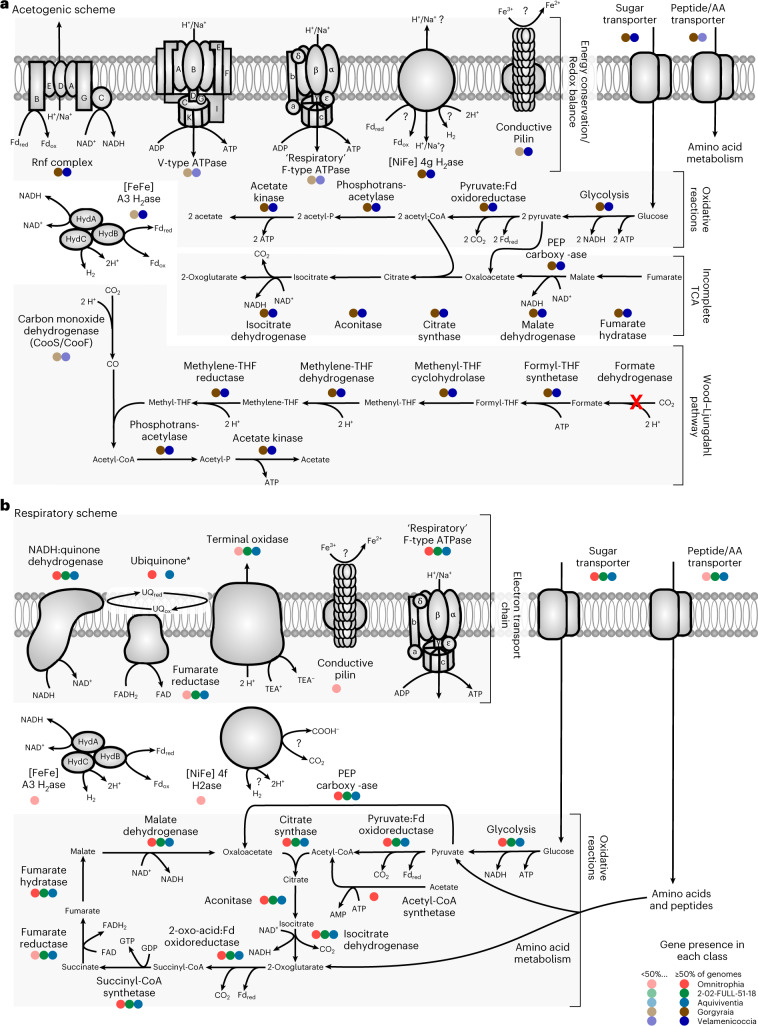
Conserved energy metabolism in major lineages of Omnitrophota. **a**, Predicted metabolism of putative acetogens or syntrophs in Velamenicoccia and Gorgyraia. **b**, Predicted metabolism of putatively respiratory lineages Omnitrophia, Aquiviventia and 2-02-FULL-51-18. Reactions are represented by arrows. Enzymes or complexes that are present are represented by coloured circles. Colours correspond to each class. Shapes are opaque if gene or gene set catalysing a given reaction is present in the representative genomes of ≥50% of species, transparent if >1% and <50% of species, or deleted if present in only one or no species. See Supplementary Tables 5 and 6 for details of these features for ANI cluster representatives. Red X indicates enzyme/complex is absent in Omnitrophota. * indicates canonical ubiquinone pathway is not complete. Source data

**Extended Data Fig. 5:**
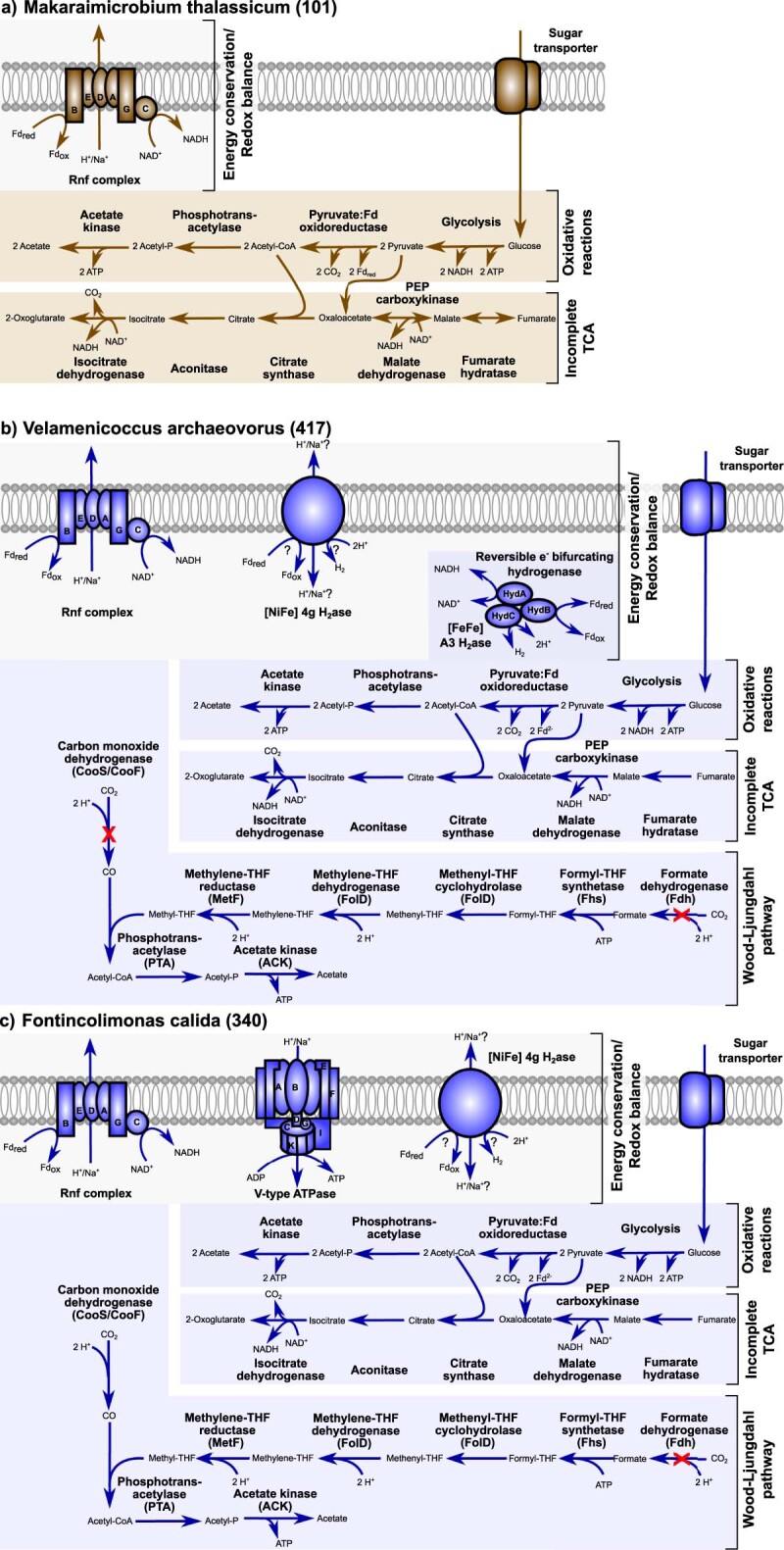
Examples of the acetogenic or syntrophic metabolism scheme in Omnitrophota genomes. Predicted energy and carbon metabolism of putatively acetogenic or syntrophic species (a) *Makaraimicrobium thalassicum*, (b) *Velamenicoccus archaeovorus*, and (c) *Fontincolimonas calida*. Lines represent genes or modules as appropriate. Reactions are represented by arrows. A red ‘X’ indicates that a complex or gene is missing or incomplete.

**Extended Data Fig. 6:**
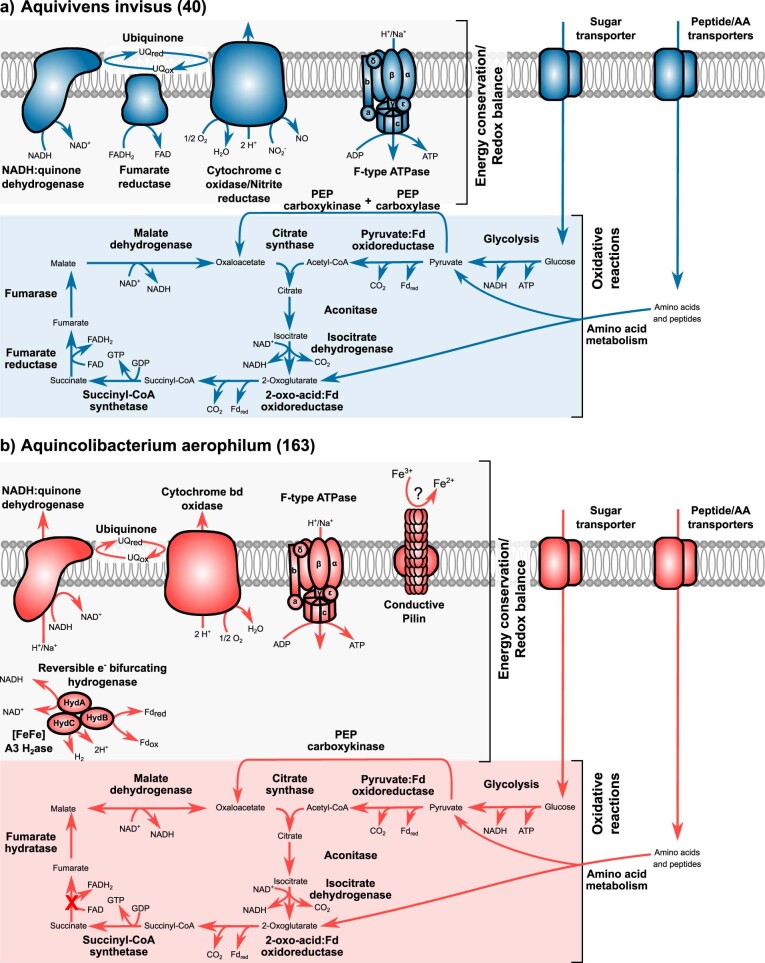
Examples of the respiratory metabolism scheme in Omnitrophota genomes. Predicted energy and carbon metabolism of putatively respiratory species (a) *Aquivivens invisus* and (b) *Aquincolibacterium aerophilum*. Lines represent genes or modules as appropriate. Reactions are represented by arrows. A red ‘X’ indicates that a complex or gene is missing or incomplete.

Genes encoding energy conservation pathways are ubiquitous in Omnitrophota genomes, and each class has either an acetogenic or respiratory scheme (Figs. 3 and 4, and Extended Data Figs. 5 and 6). Most Gorgyraia (32/39) and Velamenicoccia (73/113) genomes encode the key genes for sugar transport, Embden-Meyerhof glycolysis, ferredoxin reduction via pyruvate:ferredoxin oxidoreductase, and acetogenesis via phosphotransacetylase (Pta) and acetate kinase (Ack) (Figs. 3 and 4a). This pathway yields ATP from sugar oxidation via glycolysis and the ATP-yielding hydrolysis of acetyl-CoA to acetate via acetyl-P^24^. These genomes also encode a conserved Rnf complex that could restore NAD^+^ and oxidized ferredoxin pools^25^, or in reverse, generate an electrochemical gradient to power an ATPase^26^. PEP carboxykinase provides another possible source of ATP while simultaneously generating oxaloacetate to connect glycolysis with the biomass precursor-generating reactions of the ‘horseshoe’-type tricarboxylic acid (TCA) cycle, as previously described^7^ (Fig. 4a and Supplementary Table 6). Acetogenesis is predicted in 96 species, mapping to 7 Gorgyraia families and 8 Velamenicoccia families (Fig. 3, Supplementary Table 5 and Supplementary Fig. 10). However, these putative acetogens differ on the basis of the presence and completeness of the Wood-Ljungdahl pathway (WLP) and the types of hydrogenases (Supplementary Fig. 11) and ATPases present. Three examples of acetogenic pathways are described here. The most basic pathway, as described above, is exemplified by *Makaraimicrobium thalassicum* (Extended Data Fig. 5a) and found in 14 species in the Gorgyraia families Gorgyraeaceae, Taenaricolaceae and JABMRG01, and the Velamenicoccia family 4484-1171. A basic acetogenic pathway plus a partial WLP is exemplified by *V. archaeovorus* (Extended Data Fig. 5b). This version of the WLP lacks formate dehydrogenase (K05299, K15022), suggesting formate as a substrate for the methyl branch (Figs. 3 and 4a, and Supplementary Table 5), and AcsA and CooS/F, suggesting carbon monoxide (CO) as a substrate for the carbonyl branch. *V. archaeovorus* also encodes 4 g nickel-iron membrane-bound hydrogenase and cytoplasmic A3 iron-only hydrogenases for redox balance. This general pathway mapped to 24 species in the Velamenicoccia families Velamenicoccaceae, Profunditerraquicolaceae and DTHP01, and the Gorgyraia families FEN-1320 and CAIMPC01. Other Gorgyraia and Velamenicoccaceae species encode this same acetogenic pathway, plus CooS/F, and thus potentially fix both CO_2_ and CO. An example of this metabolism is found in *Fontincolimonas calida* (Extended Data Fig. 5c), which also encodes a V-type ATPase and a 4 g NiFe membrane-bound hydrogenase. This most complete acetogenic pathway is present in 19 species from the Gorgyraia families Aquitaenarimonadaceae, JABMQH01, JABMRJ01, JABMSB01, 2-01-FULL-45-10, RBG-13-46-9 and UBA10183, and the Velamenicoccia families Profunditerraquicolaceae, Ghiorseimicrobiaceae, DTHP01, UBA12090, QZM4-1-127 and QZM4-1-77. We note that WLP enzymes can also catalyse reverse acetogenesis, fixation of acetate to acetyl-CoA, and can ligate coenzyme A to propionate or other short-chain fatty acids^27^. Direct propionate utilization via acetate kinase (Fig. 4a) and phosphate transacetylase is consistent with the enrichment of Profunditerraquicolaceae in an anaerobic reactor community fed propionate at a high dilution rate^28^.

Unlike most Gorgyraia and Velamenicoccia, many species in Pluralincolimonadales (for example, *Pluralincolimonas frigidipaludosa*; Figs. 1 and 3) and Zapsychrales (for example, *Fredricksoniimonas* spp.; Figs. 1 and 3) lack acetate kinase and phosphate transacetylase and instead encode acetyl-CoA synthetase and diverse catabolic pathways (Supplementary Fig. 10). The four Pluralincolimonadales species encode a reversible acetyl-CoA synthetase (TIGR02717) and a simplified electron transport chain including respiratory complex I (PF00346) and an F-type ATPase (M00157). Of the 39 species clusters in the Zapsychrales, 18 encode respiratory complex I, 13 encode respiratory complex II (M00149) and 37 encode an F-type ATPase. Thirteen species of Zapsychrales encode a reversible acetyl-CoA synthetase (TIGR02717), suggesting acetogenesis or acetate utilization. The variable presence of cytochrome bd ubiquinol oxidase (M00153; 6 species), cytochrome c oxidase (M00154; 1 species) and oxidoreductases for reduction of nitrate, nitrite and metals indicate a patchwork of respiratory systems in Zapsychrales and Pluralincolimonadales, with little evidence of vertical inheritance (Fig. 3 and Supplementary Table 5). Additionally, 23 genomes encode putative reversible desulfovirdin-type dissimilatory sulfite reductases (DsrA, COG2221) (Supplementary Table 5 and Supplementary Fig. 9), which could either reduce sulfite to sulfide or oxidize sulfide to sulfur^29^, as has been suggested for SKK-01 and supported by abundant intracellular sulfur^5^.

## Diverse respiration in other classes

Omnitrophia, Aquiviventia and 2-02-FULL-51-18 lack the WLP and instead encode diverse respiratory pathways with simplified electron transport components and several possible terminal electron acceptors. Capacity for aerobic respiration via cytochrome bd ubiquinol oxidase (4 species) or cytochrome c oxidase (18 species) is highly variable, although most Aquiviventia (10/13) seem to be aerobic via cytochrome c oxidase. Denitrification genes are encoded by some species of Omnitrophia (9/23), Aquiviventia (5/13) and class 2-02-FULL-51-18 (2/5), although none encode a complete denitrification pathway (Fig. 3 and Supplementary Table 5). Some Omnitrophia (10/23) encode homologues of periplasmic cytochromes thought to be used by *Desulfovibrio ferrophilus* for dissimilatory metal reduction^30^ (Fig. 3). Some species clusters of Omnitrophia (12/23) and 2-02-FULL-18 (3/5) encode putatively reversible acetyl-CoA synthetases (TIGR02717), suggesting acetogenesis or acetate utilization in addition to respiration. Conductive pilins are predicted^31^ sparsely across species of Omnitrophia (11/23), Gorgyraia (14/39) and Velamenicoccia (49/116) (Fig. 3); pilins of this type can facilitate direct electron transfer between syntrophic partners or to mineral surfaces^32^. Two examples of respiratory metabolisms are found in *Aquivivens invisus* in the Aquiviventia (Extended Data Fig. 6a) and *Aquincolibacterium aerophilum* in the Omnitrophia (Extended Data Fig. 6b). *Aquivivens invisus* encodes transporters for sugars and amino acids, and glycolysis and TCA cycle. Respiratory complexes I and II and a ubiquinone biosynthetic pathway are present, enabling electrons to flow into the quinone pool from either NADH via complex I or FADH_2_ via complex II. Terminal electron acceptors could be oxygen, via cytochrome c oxidase, or nitrite. *Aquincolibacterium aerophilum* similarly encodes sugar and amino acid transport systems and Embden-Meyerhof glycolysis, but the TCA cycle lacks succinate dehydrogenase. Thus, it has a simplified respiratory system consisting of complex I, ubiquinone and cytochrome bd ubiquinol oxidase, suggesting a microaerophilic lifestyle. A group A3 [FeFe] electron bifurcating hydrogenase and conductive pili could also regenerate NAD^+^ and FAD via hydrogen production or metal reduction.

Although a previous report^8^ highlighted genes for methanotrophy in Omnitrophota, no evidence for methanotrophy was found in the Omnitrophota we analysed. In ref. ^10^, there was misclassification of the OLB16/SURF_12 lineage (where a particulate monooxygenase subunit homologue was observed) as ‘Omnitrophica’. This misclassification and more detail on catabolic pathways in Omnitrophota genomes are discussed in Supplementary Note 2. Additional interpretations of the physiology of Omnitrophota are discussed in Supplementary Note 3.

## Predatory or parasitic lifestyles in Velamenicoccia

**Fig. 5:**
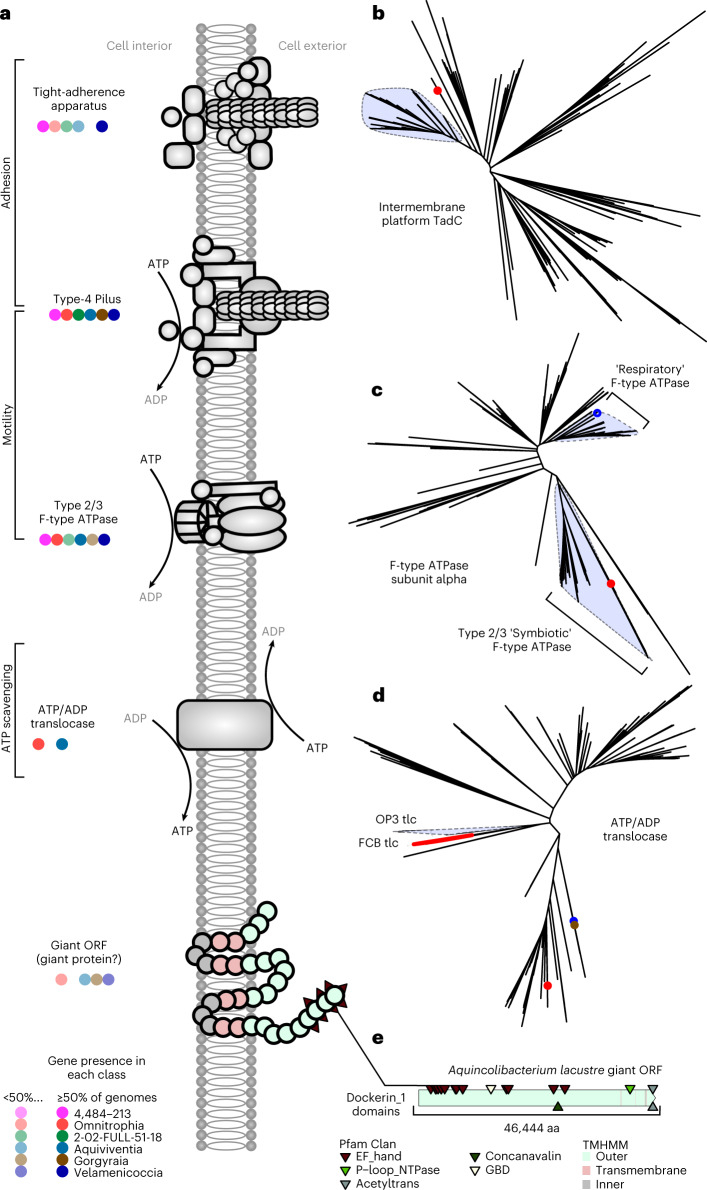
Summary of genomic evidence for parasitism and predation. **a**, Systems related to symbiosis in Omnitrophota genomes. Circles correspond to occurrence in each class, with lighter-shaded circles indicating <50% of species in the class encoding the system. **b**, Phylogeny of homologues of ‘tight-adherence’ apparatus intermembrane platform protein TadC. The highlighted clade indicates a cluster of homologues from Omnitrophota genomes. The characterized TadC from *Halobacteriovorax marinus* is indicated with a red point. **c**, Phylogeny of F-type ATP synthase subunit α. Highlighted clades indicate Omnitrophota proteins putatively involved in chemiosmotic ATP synthesis (‘Respiratory’ or ‘Symbiotic’). Putative ‘symbiotic’ ATPase gene clusters in Omnitrophota genomes are typically co-located with type-4a pilus gene clusters as in *Mycoplasma* genomes. Tips corresponding to the biochemically characterized respiratory homologue from *Waddlia chondrophila* and the pathogenesis-related homologue from *Mycoplasma mobile* are represented with blue and red points, respectively. **d**, Phylogeny of ATP/ADP translocase homologues. The light-blue highlighted clade represents homologues Omnitrophota. The red clade represents homologues from Flavobacteriaceae, including *Croceibacter atlanticus*. The red point represents a homologue from *W. chondrophila*, blue from ‘*Ca.* Babela massiliensis’ and brown from the Omnitrophota species *P. frigidipaludosa*. **e**, Illustration of the largest ORF from the class Omnitrophia, which encodes domains possibly involved in adhesion. Source data

As well as near-complete biosynthetic and energy conservation capacity, we found genes indicative of symbiotic interactions in Omnitrophota genomes (Fig. 5). These genes have diverse predicted functions, so different types of symbiosis may occur in this phylum. Predation or parasitism seems likely in the Velamenicoccia given the lifestyle of *V. archaeovorus* LiM^6^ and the conservation of genes associated with this lifestyle. For example, *V. archaeovorus* LiM encodes a complete tight-adherence (Tad) complex that is expressed during co-cultivation with *Methanosaeta*^6^ (Genome 417, GCA_004102945.1, CP019384.1, locus 12382-29832). Two or three proteins of the TadBC and RcpA complex are encoded by more than half of the Velamenicoccia species representative genomes (74/116). Phylogenetically, TadB and TadC (Fig. 5b and Supplementary Fig. 12) from Velamenicoccia group with homologues from other Omnitrophota and Bdellovibrionales. *Bdellovibrio* and like organisms (BALOs) use Tad complexes to attach to and/or enter host bacterial cells^33^. In Velamenicoccia, homologues of RcpA, the largest component of the multimeric outer membrane secretion channel, are not related to those from Bdellovibrionales, but instead group with RcpA from predatory *Stigmatella*, *Vulgatibacter* and *Lysobacter*. These relationships suggest common functionality of the Tad complex in these organisms as predators, although more complex symbiotic interactions should not be ruled out. Tad complexes are absent in genomes from Velamenicoccia families DTHP01, Fen-1317 and 4484-171, and some genomes of Profunditerraquicolaceae, suggesting potential changes in the mechanisms and/or nature of symbiosis.

*V. archaeovorus* LiM also expresses type-4a pili^6^. Type-4a pili can function in cell-cell attachment and are necessary for epibiontic predatory lifestyles^34^. Nearly all Velamenicoccia genomes (135/141) encode at least one copy of a type-4a pilus (Fig. 3 and Supplementary Fig. 10), similar to BALOs and at much higher frequencies than free-living bacteria (Supplementary Table 7). Adjacent to type-4a pilus gene clusters in many Velamenicoccia species (56/111), including *V. archaeovorus* LiM, are genes encoding a non-respiratory homologue of the F-type ATP synthase (Fig. 5c). The α-subunit of this complex is distinct from prototypical ATP-fixing genes (Fig. 5b and Supplementary Fig. 13), clustering with type-2 and type-3 F-type ATPases used by *Mycoplasma* to power gliding motility on eukaryotic cell surfaces^35^. The β-subunit of the ATPase clusters with those from other Omnitrophota, ‘*Ca.* Saccharimonadia’ (TM7)^36^ and ‘bacterium AB1_lowgc’^37^, a relative of ‘*Ca.* Dependentiae’ (TM6)^37^. The genomic architecture and phylogeny of these type-4a pili and F-type ATPases suggest a role in attachment and motility on surfaces of larger host cells during symbiosis.

Finally, giant open-reading frames (ORFs) longer than 20 kb are found in many Velamenicoccia genomes (77/116). This is an underestimate because of the incompleteness of the SAGs and MAGs. No catalytic RNA domains were found in the giant ORFs, suggesting that they may be transcribed as single mRNAs. Peptides mapping to a giant ORF were increased 3-fold in *V. archaeovorus* LiM cells attached to larger cells versus unattached cells, and it was proposed^6^ that the encoded giant protein degrades surface polysaccharides of target cells during predation. The *V. archaeovorus* LiM giant ORF codes for 39,678 amino acids and 42 predicted transmembrane helices, and was proposed to form an extracellular ‘coat’ (*Velamenicoccus* means ‘coated coccus’).

We note that most giant ORFs encoded by Velamenicoccia genomes have few annotated domains and are poorly conserved, so their functions in Velamenicoccia are difficult to assess. ORFs of similar character, albeit smaller size, have also been described in the Patescibacteria^38^ and Nanohaloarchaeota^39^, and proposed to serve as adhesins to attach to host cells and form pores in the S-layer or membrane to gain access to the cytoplasm. Large ORFs with a putative role in adhesion are also present in the genome of *Chlorobium chlorochromatii*^40^, an epibiont of ‘*Ca.* Symbiobacter mobilis’^40^. Although the nature of these giant ORFs is poorly understood, their prevalence in Velamenicoccia is consistent with a symbiotic, possibly predatory or parasitic lifestyle.

## Symbiotic lifestyles in other Omnitrophota classes

These same signatures of symbiosis occur in genomes of other Omnitrophota classes, but their distribution, frequency and evolutionary origins vary. For example, genes encoding the TadBC and RcpA complex are found at lower frequencies in other classes (Fig. 3). Phylogenetic analyses of TadBC and RcpA suggest that they have distinct evolutionary histories, but in some cases Omnitrophota homologues are closely related to those from known bacterial parasites or predators (Supplementary Fig. 12). Genes encoding most components of type-4a pili are common in all other classes (Omnitrophia (19/23), Aquiviventia (11/13), Gorgyraia (38/40) and class 2-02-FULL-51-18 (4/9)), along with genes encoding type-2 and type-3 F-type ATPases (Omnitrophia (9/36), Aquiviventia (4/13), Gorgyraia (5/40) and class 2-02-FULL-51-18 (1/5)). The latter forms a monophyletic clade of Omnitrophota proteins with evidence of horizontal gene transfer within the phylum and multiple gene loss events (Supplementary Fig. 13). In 57 out of the 66 contigs encoding both a type-4 pilus and a type-2 or type-3 F-type ATPase complex, these genes were co-located within 10 kbp (Supplementary Table 8). These ‘symbiotic’ F-type ATPase/T4aP loci were detected in every class of Omnitrophota, indicating a widespread mechanism of symbiosis in this phylum.

Giant open-reading frames (ORFs) longer than 20 kb are also common in Omnitrophia (20/23), Aquiviventia (6/13), Gorgyraia (11/40) and class 2-02-FULL-51-18 (1/5) (Fig. 3). Most giant ORFs encoded by Omnitrophia include multiple predicted transmembrane helices with large predicted extracellular domains including those for cell adhesion^41^. For example, ORFs from *Multiplicimicrobium inquinatum* (Genome 171) and *Omnitrophus fodinae* (Genome 190) encode discoidin (PF00754) domains and ORFs from *M. inquinatum* and SAG-3300015153 (Genome 196) encode laminin_G_3 (PF13385) domains (Supplementary Fig. 14). Similarly, a giant ORF from *Aquinicolibacterium lacustre* (Genome 157) has nine non-cellulosomal dockerin (PF00404) domains that may serve as adhesins^42^ (Fig. 5). However, adhesins are less common in giant ORFs of the other classes and overall, few annotated domains are present in these giant ORFs, obscuring their functions. The implication that Omnitrophota may use giant proteins for cell-cell adhesion suggests a broader context for giant ORFs in the otherwise reduced genomes of bacterial symbionts. However, the roles of these giant ORFs are poorly understood.

An additional symbiosis factor, ATP/ADP translocase (K03301), was identified only in genomes of Aquiviventia (11/31) and Omnitrophia (20/36) (Figs. 3 and 5d, and Supplementary Fig. 15). Translocases of this type are used by intracellular parasites in the Rickettsiae and Chlamydiota to import cytoplasmic ATP while parasitizing a host^43^ and are common in BALO genomes (Supplementary Table 7). The translocases from Omnitrophia and Aquiviventia form a well-supported, monophyletic cluster related to those from uncultured Flavobacteriaceae, including the epibiontic symbiont of diatoms, *Croceibacter* *atlanticus*^44^. A single homologue encoded by *P. frigidipaludosa* in the Gorgyraia is related to a putative ATP/ADP translocase from ‘*Ca.* Babelia massiliensis’^45^ (an obligate intracellular parasite of *Acanthamoebae* belonging to ‘*Ca.* Dependentiae’^46^), and both homologues are basal to known Chlamydiota ATP translocases. These ATP/ADP translocases may play a role in scavenging cytoplasmic ATP during symbiosis.

**Extended Data Fig. 7:**
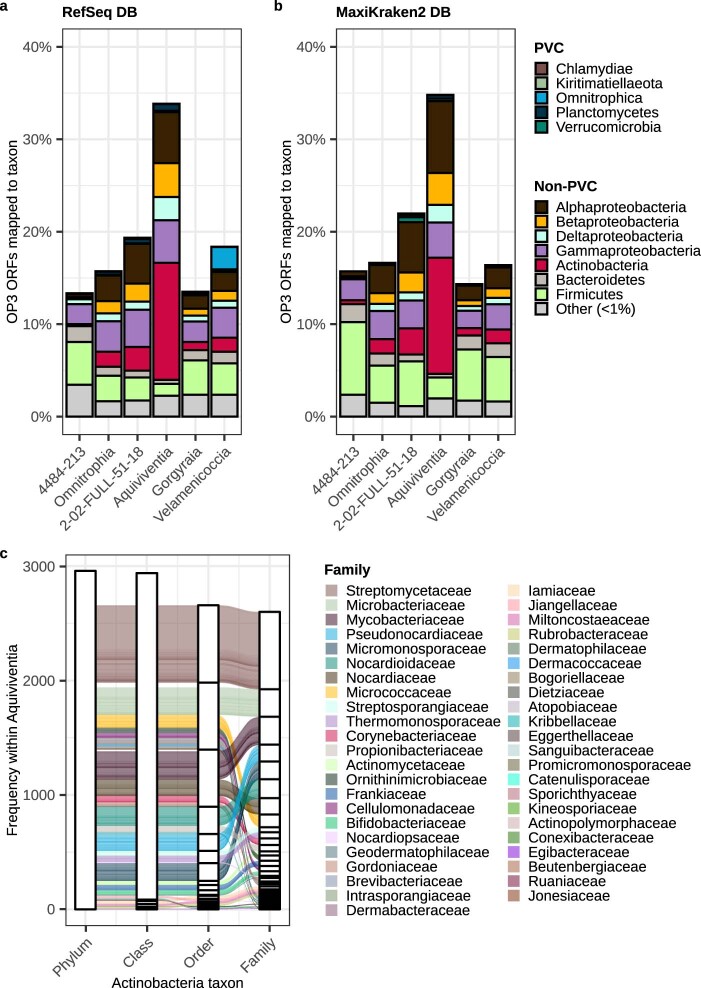
Taxonomic classification of Omnitrophota ORFs. Taxonomic affiliation of Omnitrophota ORFs according to classification using Kraken RefSeq (a) and MaxiKraken (b) databases. *Velamenicoccus archaeovorus* is represented in RefSeq, and as a consequence, closely related ORFs from Velamenicoccia genomes classify as ‘Omnitrophica’. (c) Family-level affiliation of ORFs from Aquiviventia genomes. Source data

Taken together, the presence of complete biosynthetic and energy conservation pathways, and several systems suggesting symbiosis, imply complex lifestyles perhaps involving symbiotic and free-living phases, as has been proposed for Patescibacteria and DPANN archaea^47^. Given the conservation of genes related to predation in most Velamenicoccia, we suggest that most Velamenicoccia may be epibiontic predators, as exemplified by *V. archaeovorus*^6^. *V. archaeovorus* LiM was observed attaching to and predating on a variety of cells in a methanogenic limonene-degrading culture^6^. However, LiM cells were also frequently found as free unattached cocci but with low ribosome count, suggesting low rates of anabolism. This observation, and the biosynthetic capacity and energy conservation pathways encoded by Velamenicoccia genomes suggest that these organisms might persist in nature as individual cells, especially given the low maintenance energy associated with small cell size. This interpretation is consistent with our recovery of 31 SAGs from groundwater and anoxic lakes via FACS exclusively as small cells, with no co-sorts of Velamenicoccia or any other Omnitrophota with other cells. Which, if any, Omnitrophota have specific interactions with host species or genera is unknown; however, Aquiviventia genomes shared a disproportionate fraction of genes of actinobacterial origin, especially Streptomycetaceae, suggesting a possible partnership (Extended Data Fig. 7).

Despite the abundance of genes suggesting predation or parasitism within Omnitrophota, most Gorgyraia lack Tad complexes and ATP/ADP translocase genes (Fig. 2), suggesting that free-living lifestyles might be more common in this class. In support of this, SKK-01 (ref. ^5^) has the only confirmed large cells in the phylum, and many Gorgyraia were retained on large-size filters (>0.65 μm; Fig. 2). However, serial filtration and FACS indicated both large and small cell sizes in the class, and the scattering of systems indicating possible symbiosis suggests a complex history for this class.

## Stable isotope incorporation in Omnitrophota

**Fig. 6:**
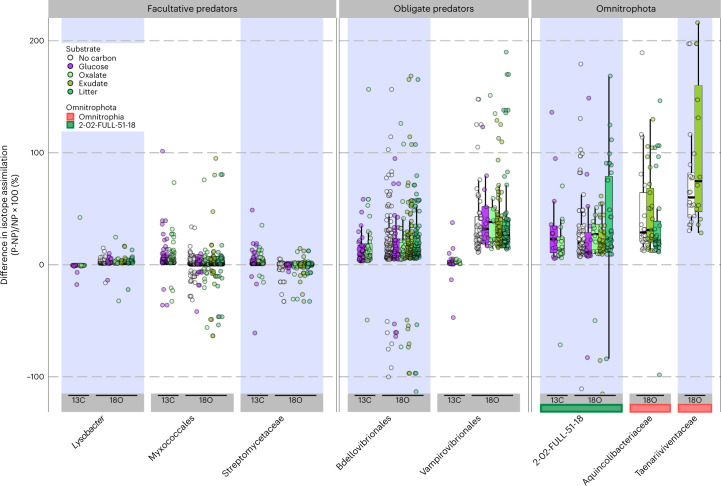
Family-level qSIP in diverse soils. *Y* axis shows the percent difference between AFE ratios for a given taxon (P) compared to all non-predatory (NP) taxa from the same sample. Boxes display the median and inner quartiles, while whiskers extend to the 95% confidence interval of the distribution of AFE ratios for a given taxon within each experimental group. *N* = 114 qSIP experiments. Source data

Bacterial predators, especially obligate predators, incorporate labels in stable-isotope probing experiments faster than those with other feeding behaviours^48^. To test the hypothesis that some Omnitrophota are highly active in natural environments, existing quantitative stable-isotope probing (qSIP) data were analysed to focus on Omnitrophota. qSIP experiments in three geologically distinct oxic soils derived from andesite, basalt or granite weathering revealed high intrinsic ^18^O-H_2_O incorporation levels by the families Aquincolibacteriaceae and Taenariiviventaceae (class Omnitrophia) and the family 2-02-FULL-51-18 (class 2-02-FULL-51-18) (Fig. 6). Since the ^18^O from water exchanges with ^16^O atoms in free nucleotide and nucleoside pools, but not DNA, these high ^18^O values are consistent with high rates of DNA synthesis and/or high rates of consumption of biomass from labelled cells, either through predation, parasitism or necromass consumption. An alternative interpretation is that diverse Omnitrophota are stimulated by the addition of diverse organic compounds directly, but we consider this unlikely because ^18^O incorporation values from these Omnitrophota were not significantly different from those of obligate predators in the Bdellovibrionales and Vampirovibrionales in the same dataset (*P* > 0.05, ANOVA with post-hoc Tukey’s HSD) but were higher than those of facultative predators such as *Lysobacter*, Myxococcales and Streptomycetaceae, and free-living bacteria en masse (*P* < 0.05, ANOVA with post-hoc Tukey’s HSD; Supplementary Fig. 16); thus, we describe them as hyperactive. We caution against the interpretation that these Omnitrophota are necessarily obligate predators because their small cell size, and therefore higher DNA/biomass stoichiometry, might contribute to higher ^18^O content of Omnitrophota and other small cells. However, high isotope incorporation of facultative predators with large cell size in the same datasets and in other soils^48^ argues that the overall isotope incorporation pattern observed here for Omnitrophota is due to some form of symbiosis. Family 2-02-FULL-51-18 also assimilated high amounts of ^13^C-labelled glucose and oxalate, although long incubation times and high soil community complexity complicate interpretation of carbon source utilization.

These high isotope incorporation values, along with those previously observed in Black Sea sediments^9^, mean that high metabolic activity and/or consumption of labelled cell mass have been reported in four of the six classes of Omnitrophota in both anoxic sediments (Black Sea sediments^9^, Velamenicoccia and Gorgyraia) and oxic soils (diverse soils, Omnitrophia and class 2-02-FULL-51-18). These patterns of substrate utilization in oxic soils and anoxic sediments align with metabolic and ecological predictions made here. The classes Velamenicoccia and Gorgyraia—predicted to be predominantly obligately anaerobic acetogens (Fig. 4)—were highly active in anoxic Black Sea sediments^9^, but they were present at very low abundance in the soils studied here and did not incorporate isotopic substrates. Conversely, aerobic respiration is predicted (Fig. 4) for many members of the Omnitrophia families Aquincolibacteriaceae and Taenariiviventaceae and class 2-02-FULL-51-18, and members of these families were indeed highly active in oxic soils but not in anoxic Black Sea sediments.

## Discussion

Our analysis of a compendium of Omnitrophota genomes reveals that this enigmatic bacterial phylum is diverse, ubiquitous and has the capacity for a free-living chemoheterotrophic or mixotrophic lifestyle, with genomic markers consistent with parasitism and predation. The environmental distribution of Omnitrophota precludes them from being pathogens of macroscopic hosts. If Omnitrophota are symbiotic, at least during some stages of a complex lifestyle, then potential hosts and mechanisms of symbiosis may be lineage specific.

Given the known host-associated lifestyle of *V. archaeovorus* LiM and the highly conserved nature of predation-related genes, we suggest that host dependency may be common in Velamenicoccia. Conversely, the variable presence of predation-related genes and the specific presence of ATP/ADP translocases mean that Omnitrophia and Aquiviventia may have different modes of symbiosis. The high isotope incorporation values in Velamenicoccia, Gorgyraia, Omnitrophia and class 2-02-FULL-51-18 are consistent with this interpretation. Direct experimental evidence will be needed to test this intriguing hypothesis in future studies.

It is important to note that another group of nanobacteria, the Patescibacteria, that is widely recalcitrant to laboratory cultivation has been labelled as obligate parasites^49^ or as free-living cells^22^. Key to the latter interpretation was the lack of cell-cell associations detected by FACS during integrated FACS and single-cell genomics. This approach has detected specific associations between *Nanoarchaeota* and archaeal hosts^50,51^ in geothermal environments, and between ‘*Ca.* Saccharibacteria’ and actinobacterial hosts^52,53^. The absence of evidence for cell-cell associations between Omnitrophota and any other species (*n* = 109 Omnitrophota cells) may suggest either that Omnitrophota cell-cell interactions are weak, that cells are mostly free-living, or that Omnitrophota persist in the environment as free-living nanobacteria that are either facultatively or obligately symbiotic with a free-living phase. Obligate symbiosis with a free-living phase has been proposed for *V. archaeovorus* LiM^6^, Patescibacteria and DPANN archaea^38,39^, which do not show significant cell-cell interactions in the integrated FACS and single-cell genomics pipeline used here (*n* = 770 Patescibacteria cells and *n* = 113 DPANN cells^22^). All of these possibilities are consistent with the largely complete biosynthetic and energy conservation potential of Omnitrophota.

Understanding energy conservation pathways and possible lifestyles in the Omnitrophota may enable cultivation. The analytic framework of our study has uncovered a better description of the biology of these organisms and the roles they have in Earth’s biomes.

## Methods

### Compilation of MAGs and SAGs

Genomes used in this study and their provenance are summarized in Supplementary Table 1.

Newly generated MAGs originated from geothermal environments (Tengchong, Yunnan Province, China; Beatty, Nevada, USA; British Columbia, Canada; and Guaymas Basin, Mexico), freshwater lakes (Powell Lake, British Columbia, Canada; Lake Kivu, Rwanda; Lake Alinen Mustajärvi, Finland) and wastewater (Apeldoorn, the Netherlands).

Sediment samples from hot springs in Tengchong, China were frozen before DNA extraction using the Powersoil DNA isolation kit (MoBio) or FastDNA SPIN kit (MP Biomedical). Approximately 30 Gbp (2 × 150 bp) was generated per sample using the Illumina HiSeq 4000 Platform with a 350 bp insert library at Beijing Novogene Bioinformatics Technology. Raw reads were quality filtered by eliminating adapter-contaminated reads, deleting PCR-generated duplicated reads, removing reads with an excess of ‘Ns’ (≥10% of the read) and trimming reads with a quality score of less than 15 at the 3′ end. The high-quality reads of each sample were de novo assembled individually using metaSPAdes^54^ v3.9.04 with the following kmers: -k 33, 55, 77, 99, 111. Reads were mapped to scaffolds using BBMap v38.8518. Genome binning was conducted on scaffolds with length >2.5 kbp using MetaBAT2 (ref. ^55^). The estimated quality of binned MAGs was evaluated using CheckM^56^, and initial classification was done using the GTDB Toolkit^16^ v1.1.010 to identify MAGs belonging to Omnitrophota.

Other MAGs were sequenced at the US Department of Energy Joint Genome Institute (JGI) as part of the Microbial Dark Matter II (MDM II) project. Metagenomic DNA was sequenced on the Illumina HiSeq-2500 platform (libraries with 300 bp inserts) at the JGI in 2 × 150 bp mode. Reads were trimmed and screened for laboratory contaminants with BBTools v37 (ref. ^57^ http://bbtools.jgi.doe.gov) and the sequencing errors were corrected by bfc^58^ v181 with the parameters: ‘-s 10 g -k 21’. Mate-pair reads were assembled using SPAdes^59^ v3.10.0 with kmers 21, 33, 55, 77 and -meta flag. MAGs were created by combining initial sets of genome bins from seven different binning approaches: (1) MaxBin^60^ v1.4.5 using the universal 40 marker gene set and (2) the 107 marker gene set; (3) MaxBin^61^ v2.2.4 with default parameters; (4) MetaBAT1 (ref. ^62^) v0.32.5 using the ‘super-specific’ parameter and (5) ‘super-sensitive’ parameter; (6) MetaBAT2 (ref. ^55^) v2.12.1 using default parameters; and (7) CONCOCT^63^ v0.4.0 using default parameters. All binning methods used a minimum contig size of 3,000 bp. Bins generated using the seven methods were used as input to DAS Tool^64^ v1.1.0, which was run with default parameters to generate the final MAG set.

In addition to newly generated MAGs, 316 additional genomes were collected from public data repositories: 77 public genomes from Rifle Creek, Colorado, USA (Supplementary Table 1), constituting the largest portion of Omnitrophota genomes from a single origin, and a further 239 SAGs and MAGs from published metagenomic studies conducted around the world (Supplementary Table 1). Genomes were included if they were classified as Omnitrophota in at least one version of GTDB or were a search result for ‘Omnitrophica’ or ‘OP3’ on GenBank or IMG. Many of these assemblies were duplicated between IMG and GenBank, so assemblies were dereplicated by removing sequence names from each fasta file, calculating sha256 hashes of the resulting unnamed sequence files and retaining files with unique hashes. ORFs in dereplicated assemblies were reannotated using Prodigal^65^ v2.6.3. Finally, assemblies were discarded if they did not classify into either ‘Ratteibacteriota’ or Omnitrophota according to the modified GTDB-Tk^16^ classification step described below.

SAGs originated from deep subsurface aquifer waters from Beatty (Nevada, USA), Crystal Geyser (Utah, USA) and Free State (South Africa), and sediments from Etoliko Lagoon (Greece). Samples were amended with sterile 5% glycerol and 1 mM EDTA (final concentrations) and stored at −80 °C. SAG generation and sequencing were performed by the Bigelow Laboratory for Ocean Sciences Single Cell Genomics Center (SCGC) and JGI as previously described^22,66^. Briefly, cells were stained with SYTO-9 (Thermo Fisher), separated using FACS, lysed using a combination of freeze-thaw and alkaline treatment, and genomic DNA was amplified using WGA-X in a dedicated clean room. During FACS, cell size was estimated using calibrated index FACS^66^. SAGs were subjected to low coverage sequencing (LoCoS; ∼300 k paired-end reads per SAG)^66^, assembled utilizing SPAdes 3.9.0 (ref. ^59^), as previously described^66^, and the quality of the assembled genomes (contamination and completeness) was assessed using CheckM^56^ and tetramer frequency analysis^67^. Omnitrophota SAGs were combined into 48-library pools and shipped to JGI for deeper (post-LoCoS) sequencing with NextSeq 500 (Illumina) in 2 × 150 bp mode. Read-quality filtering was performed with BBTools v37, read normalization with BBNorm and error correction with Tadpole^68^. The resulting reads were assembled with SPAdes^69^ (v3.9.0, –phred-offset 33 –sc -k 22,55,95 –12), and 200 bp was trimmed from the ends of assembled contigs, after which contigs with read coverage <2 or <2 kbp in length were discarded. SAGs were reannotated, where relevant, following procedures outlined by IMG standard protocols^70^.

### Quality control of MAGs and SAGs

The quality of each genome was assessed using a modified CheckM^56^ pipeline and CheckM2. Some of the marker genes that are systematically absent from the PVC superphylum are conserved and in single copy in many bacteria. These markers have seen widespread use in completion and phylogenomic marker analyses, including the general bacterial marker sets used to predict assembly completeness by CheckM. Consequently, the lineage workflow within CheckM may slightly underestimate completeness of Omnitrophota assemblies. This discrepancy is most evident in the genome of *V. archaeovorus* (GCA_004102945.1)^6^, which reports a lineage workflow completion estimate of only 92% based on a general bacterial marker set, despite the genome being sealed and therefore complete. Still, most of the markers in the general bacterial marker set were present consistently across the phylum. To provide a more accurate quality estimate, the general bacterial marker set was modified to account for a few systematically missing markers. Three markers, TIGR03594, PF13603.1 and PF02978.14 were excluded. PF02978.14 was present in only one genome. The other two markers were infrequently present and the mean phylogenetic distance between taxa with the marker was lower than the other markers within the set, indicating that, with respect to the phylum, the presence of the markers was phylogenetically constrained (Extended Data Fig. 1). Completeness and contamination were recalculated using a general bacterial marker set excluding these markers. This recalculated completeness estimate was used downstream wherever completeness estimates were required, and to determine which genomes would be selected as species representatives.

To encourage accurate completeness estimates in future Omnitrophota assemblies, we extended this approach to find other PVC-group single-copy marker genes that occur with a similar pattern to the marker set used to calculate genome completeness and contamination. Marker sets for Planctomycetota, Verrucomicrobiota and Chlamydiota were individually extracted from the CheckM reference data. An auxiliary marker set was generated from the union of the PVC-group marker sets. Omnitrophota genomes were then probed for markers within the PVC-union set. Single-copy marker genes were identified where the number of occurrences across all ANI cluster representatives was within the 95% confidence interval of the difference between completeness and contamination in those same genomes. Using this method, 122 putative single-copy marker genes were identified in Omnitrophota assemblies. These markers are provided in Supplementary Table 10.

### Species delineation, genome-based phylogeny and Omnitrophota classes

Genomes greater than 50% complete and less than 10% contaminated were considered medium quality, while those greater than 90% complete, with less than 5% estimated contamination and that contained 23S, 16S and 5S rRNA genes and at least 18 transfer RNAs were considered high quality^11^. Those 10–50% complete and less than 5% contaminated were considered low quality but were retained for phylogenetic placement. Those less than 10% complete or greater than 10% contaminated were removed from the dataset. This quality control process produced a dataset that contained 72 SAGs and 349 MAGs. Genomes were grouped into species on the basis of their membership in single-linkage clusters, with a threshold of 95% ANI according to FastANI^10^. If a high- or medium-quality representative was available, the most complete genome in each cluster was used for phylogenetic analyses. Clusters were classified to the genus level if their members included a genome present in the GTDB^12^. Genomes lacking a representative in GTDB were classified using GTDB-Tk^16^ with the r202 reference. The classification step of GTDB-Tk was modified to call a custom script that constrains phylogenetic placement to the subtree one node above the most recent common ancestor (MRCA) of Omnitrophota (p__Omnitrophota) and ‘Ratteibacteriota’ (r202: p__Ratteibacteria). Pplacer^71^ was used to place genomes on the subtree using the same reference package model parameters as the full tree but using the reduced alignment. The subtree was then trimmed by one node and grafted to replace the MRCA of the Omnitrophota and ‘Ratteibacteriota’ on the reference tree. These steps were taken to reduce the memory requirement for phylogenetic placement onto the GTDB-Tk reference tree and to exclude genomes outside of these two lineages. The classification step then proceeded as normal, using the subtree-grafted reference tree. Conserved marker gene alignments were used to construct the phylogeny of Omnitrophota. The full-length bac120 alignment^12^ was obtained from GTDB-Tk. The bcg110 marker set was generated using bcgTree v1.1.0 (ref. ^14^). Additional alignments of 56 universally conserved COGs^15^ were identified using hmmer v3.3.1 (ref. ^72^). Up-to-date bacterial core genome (UBCG) marker genes were identified and aligned using the UBCG software^13^ v3. UBCG and Bac120 marker alignments were reduced using the gappyout function of trimal^73^ v1.4.rev22. Phylogenetic trees were constructed from reduced alignments using IQ-Tree^74^ v1.6.8. Individual gene sets were aligned using mafft^75^ v7.453, then reduced using the gappyout function of trimal^73^. Substitution model testing was performed using IQ-Tree’s ModelFinder^76^, restricted to WAG, LG, JTT, JTTDCMUT and PMB. Node support was based on 1,000 SH-like aLRT (alrt) test and 1,000 ultrafast bootstrap^77^ rounds. Bac120 alignment sequences with greater than 1,000 residues in the alignment corresponding to retained low-completeness assemblies were masked using the same filter produced by gappyout. These sequences were then placed onto the Bac120 marker set tree using epa-ng^78^ v0.3.8, using a custom script to generate a reference package from IQ-tree outputs. The GTDB taxonomy was refined on the basis of the Bac120, UBCG and BCG marker set trees to find concordance between topologies. AAI was calculated between species using the procedure from ‘aai.rb’ of the enveomics^79^ script collection, reimplemented in R. Genus-level assignments were modified or added where no taxonomy existed to produce consistent intra- and intergenus AAI values in each family. Phylogenetic trees were rendered using the R package ggtree^80^.

The class-level taxonomy of Omnitrophota varies greatly between versions of the GTDB. Previous versions of the GTDB report upwards of six distinct classes, while release r202 reports only three: Omnitrophia, 4484-214 and Koll11. The problem with the Koll11 grouping is that, according to the GTDB r202 reference tree, the base of the Koll11 lies along a large polytomy at the root of Omnitrophota; the split that separates the Omnitrophia and 4484-214 from the Koll11 is the same distance from the root as the base of the phylum itself. The taxon Koll11 is therefore invalid, since the node that forms the base of this taxon is topologically indistinguishable from the parent node of the phylum. Moreover, Koll11 is paraphyletic according to the BCG110 marker phylogeny. To enforce monophyly across the three marker phylogenies, and to ensure that the root of each class retained bootstrap support within the Bac120 tree, the Koll11 class was split into the four other classes: 2-02-FULL-51-18, Aquiviventia, Gorgyraia and Velamenicoccia. The splintering of Koll11 was required to resolve multiple phylogenetic paradoxes that arise when considering other marker sets. While a relationship among 2-02-FULL-51-18, Aquiviventia, Gorgyraia and Velamenicoccia is supported by Bac120 and UBCG trees, the BCG110 phylogeny reports a supported grouping with 2-02-FULL-51-18 and Omnitrophia. This indicates that, at the very least, 2-02-FULL-51-18 should be excluded from grouping with the other Koll11 genomes. However, according to the UBCG phylogeny, 2-02-FULL-51-18 and Aquiviventia form a supported grouping together. It follows that if the members of 2-02-FULL-51-18 form a distinct lineage, then the members of Aquiviventia must be one as well. With these two taxa parsed out, the next possible split for the base of a class lies at the MRCA of Gorgyraia and Velamenicoccia. While this node is monophyletic according to all marker trees, it lacks bootstrap support on the Bac120 tree. Therefore, we cannot confidently say that this node represents the parental node of a major lineage. Nevertheless, these taxa aim to hit a moving target; the addition of more data—more genomes from organisms closely related to the long-branched orphans that confound many phylogenetic methods—may one day support a class or ‘superclass’ including the Velamenicoccia, Gorgyraia and some of the ‘incertae sedis’ orders previously encapsulated by Koll11.

### Genome annotation

Gene function annotation was performed using kofamscan (DB v2020-02-02)^81^, txsscan^82^, METABOLIC^83^, FeGenie^30^, TMHMM^84^ and hmmer^72^. The hmmsearch function of hmmer 3.3.1 was used to annotate a file containing all predicted protein sequences from Omnitrophota genomes. For PFAM and TIGRFAM annotation libraries, the noise cut-off was used as a threshold for a positive result. For kofamscan, METABOLIC and FeGenie annotations, score thresholds were applied according to the datafiles included with each software package. To further classify hydrogenases, protein sequences annotated as putative hydrogenases by METABOLIC were reannotated using the HydDB^85^ web-interface. Putative electrically conductive pili were identified on the basis of the aromatic content of the predicted amino acid sequence, as described previously^86^. 16S and 23S rRNA gene sequences were identified and extracted using Metaxa2 (ref. ^87^) v2.2.1. COGs were annotated using rpsblast v2.9.0 (ref. ^88^) against COG position-specific scoring matrices provided by the National Center for Biotechnology Information taxonomy (NCBI) CDD database with an *e*-value threshold of 1 × 10^−2^. tRNAs were annotated using tRNAscan-SE^89^ v2.0.7. Other RNA motifs were annotated using the Rfam database^90^ v14.5 with Infernal^91^ v1.1.4.

### Omnitrophota genome size estimation and comparison

Complete genome size was estimated for each Omnitrophota genome using the formula*:* Size_est_ = (Size_obs_ − (Size_obs_ × contamination))/(completeness), where Size_obs_ is the total size of the assembly, and contamination and completeness are genome quality estimates calculated from single-copy marker genes in a general bacterial marker set excluding markers systematically absent from the phylum (Supplementary Fig. 2). Genome sizes were also predicted using this approach for all type species genomes in the GTDB, considering only phyla with >4 members. Additionally, some phyla, such as Firmicutes (labelled Firmicutes_A, Firmicutes_B and so on in GTDB) were collapsed into single units for simplicity. All phylum pairs and Omnitrophota class/phylum pairs were compared using ANOVA followed by Tukey’s HSD. The quantile of each Omnitrophota genome was then calculated as a function of this genome collection.

### Nomenclature and avoiding synonymy

Omnitrophota taxa were named following the rules of the SeqCode and registered in the SeqCode Registry^18^. In most cases, decisions on nomenclature were straightforward; however, a few special cases are described here. Historically, three *Candidatus* names have been proposed for Omnitrophota genomes included in this study. These were considered and modified both to follow the SeqCode and to limit intersection with existing names. Previous names for the phylum have been ‘Omnitrophica’^4^ or ‘Omnitrophicaeota’^1^, but Omnitrophota is adopted here to follow the SeqCode. In 2017, the genome for *V. archaeovorus* LiM was published using the name ‘*Ca.* Vampirococcus archaeovorus’ LiM and was later revised^6^ to differentiate the genus from two unrelated previously published *Candidatus* genera of that same name^38,92^. The genus name *Omnitrophus* was given priority over other names and the root was used to name higher taxa, including the phylum. The name ‘*Ca.* Omnitrophus magneticus’ SKK-01 (ref. ^5^) conflicts with the older name *Omnitrophus fodinae*; the representative genomes of these species belong to different classes. In addition to this, the genome of SKK-01 (GCA_000954095.1) suffers from assembly problems: the assembly contains 23 short (<1,000 nt) contigs, each of which encode a (typically single-copy^56^) tRNA-arginyl synthetase (PFAMs PF03485 and PF00750). Therefore, we recommend disuse of the name ‘*Omnitrophus magneticus*’ and do not propose an alternative here. On the other hand, the genome of *Omnitrophus fodinae* has low genome completeness (for example, 65.01% with CheckM2) and normally would not be sufficient for a nomenclatural type under the SeqCode. However, no other genomes assigned to the genus are better, so we chose to retain this name, with the genome assembly (GCA_000405945.1) as the nomenclatural type for the species.

As a large number of names were generated, special care was taken to identify and mitigate collisions between validly published and *Candidatus* taxonomic names and the new names proposed here. To do this, a custom algorithm was implemented to check every proposed taxon against entries in the Catalogue of Life^93^, the NCBI database^94^, the Interim Register for Marine and Nonmarine Genera^95^ and the list of *Candidatus* taxa^96^. The algorithm first searches for exact matches within these data sources, then uses an Rcpp implementation (10.6084/m9.figshare.3386308.v1) of the Damerau-Levenshtein (DL) distance algorithm to identify taxon names with an edit distance of 2 or less from an existing taxon. More than 450,000 genera are defined between these sources, so an optimization of the DL algorithm was used to shorten runtime (Supplementary Fig. 5).

The Damerau-Levenshtein distance algorithm calculates the minimum number of edits that must be made to transform one string into another. An edit is the substitution, insertion, deletion or transposition of characters in the string. To determine this distance, the DL algorithm calculates a pairwise alignment between strings—a computationally intensive operation that can become cumbersome to perform over a large dataset. By specifying a maximum distance, *D*, the operation can be reduced to consider only distances that will be less than or equal to *D* according to less complex algorithms; for any comparison between strings *a* and *b* where the DL distance is less than *D*, two criteria must be satisfied:

1. The difference in the character lengths of *x* and *y* will be less than or equal to *D*. Every addition or deletion of a character counts as an edit towards the overall distance between two strings. Because of this, if the number of edits required to match the string lengths, regardless of their content, exceeds the maximum allowable distance, then the comparison can be disregarded. This operation is a simple difference between two integers and is much faster than the overall DL distance algorithm.
2. For comparisons where the first criterion is satisfied, the number of unique characters present in *a* that are not in *b* will ‘not’ be greater than *D*. Each new character added in the process of transforming one string into another requires a single edit whether by addition or by substitution. The number of edits required to add or remove these exclusive characters must not exceed *D* for comparisons with an edit distance less than or equal to *D*. This operation largely consists of the difference between two sets and is still much faster than performing the DL algorithm.

### 16S rRNA gene phylogeny and Qiime2 classifier build

Omnitrophota 16S rRNA genes were aligned against the SILVA 99% identity nonredundant 16S rRNA gene (SILVA 99nr) database^1^ v138 using the arb-silva.de web-interface ACT (alignment-classification-tree) tool. Any residues outside of the aligned region were removed. Aligned sequences were combined with an additional 657 aligned 16S rRNA gene sequences from Omnitrophota and neighbouring phyla exported from the same database. Aligned sequences with fewer than 1,000 unambiguous residues in the alignment or those with a sequence, alignment or quality lower than 75 were omitted. To limit redundancy within the alignment, sequences were dereplicated: for outgroup phyla, one sequence belonging to each genus was retained, while sequences belonging to Omnitrophota were clustered at a 99% identity threshold using vsearch^97^ v2.18.0. Sequences were then filtered according to the Lane mask in Mothur^98^. Duplicated sequences were omitted at this point. A phylogenetic tree was constructed from the masked sequences using IQ-Tree^74^ v1.6.8. Sequences within 99% identity clusters were considered to be from the same species group. Taxonomy was assigned to nodes on the phylogenetic tree on the basis of the consensus of each node’s children. This taxonomy, along with the unmasked versions of sequences represented on the phylogenetic tree (Supplementary Table 11), was then imported into Qiime2 (ref. ^19^) v2020.8. The sequences were clipped to the V4 region using the feature-classifier^99^ plugin from Qiime2 by using the EMP 515F/806R^100^ primers as parameters. A naïve-Bayesian sequence classifier was generated from the clipped sequences and corresponding taxonomy using the feature-classifier^99^ plugin from Qiime2. 16S rRNA gene distances were calculated using Mothur^98^ v1.44.3.

### EMP meta-analysis

EMP^20^ biom files were downloaded from the EMP ftp server (ftp://ftp.microbio.me/emp/release1). Sequence variants were trimmed to 90 nt and dereplicated to 3,664,846, then classified according to the SILVA 99nr database^1^ v138 using the classifier obtained from the developers of Qiime2 (ref. ^101^). Sequences classified as Omnitrophota (in SILVA: Verrucomicrobia;Omnitrophia) were classified once more using the Omnitrophota-specific classifier created here, yielding 29,249 Omnitrophota sequence variants (SV). SVs unclassified at the domain level were removed. Sequence variant tables corresponding to samples from release 1 of the EMP dataset, excluding negative controls or blanks, were merged into a single table. This table was used to calculate the environmental and geographic distribution of Omnitrophota.

### Tandem filtration and 16S rRNA gene amplicon analysis

Spring water was filtered using serialized 1 µm, 0.45 µm and 0.2 µm polyethersulfone membrane Sterivex-GP pressure filters (Millipore Sigma) through Masterflex LS-24 platinum-cured silicone tubing (Cole-Parmer) with a Geopump peristaltic pump (Geotech). The inlet tube was placed as close to the spring source as possible. Filters were purged of water, frozen immediately and kept on dry ice. The filters were transferred to a −80 °C freezer until DNA was extracted. Membranes from the 0.45 and 0.2 µm filters were pulverized manually. DNA was extracted from the membrane pulp using a FastDNA SPIN kit for soil (MP Biomedicals) according to the manufacturer’s instructions. DNA extracts were submitted to MrDNA (www.mrdnalab.com, MR DNA) for sequencing on the Illumina MiSeq platform. The updated bacterial- and archaeal-specific 515F/806R primer set was used to amplify the V4 region of the 16S rRNA gene^102,103^. 16S rRNA gene amplicon reads from serial filtration experiments were processed using Qiime2 (ref. ^99^) v2020.8. Paired-end reads were quality filtered using the quality-filter plugin of Qiime2 at default settings. Quality-filtered reads were then joined and trimmed to 150 nt, and SVs were identified using deblur within Qiime2. SVs were then classified using both classifiers as previously described.

### Phylogenetic analysis of Omnitrophota physiology proteins

Phylogenetic analyses of energy metabolism and Tad apparatus genes were carried out using a custom pipeline. Assemblies were annotated using kofamscan (DB v2020-02-02)^81^ and txsscan^82^ as described previously. Omnitrophota protein sequences annotated by txsscan as encoding Tad apparatus orthologues were retained. Omnitrophota protein sequences putatively encoding F-type ATPase subunits or the ATP/ADP translocase were retained, depending on kofamscan annotation. Reference sequences were acquired from the UniProt database using protein sequence accession codes from the KEGG database encompassing the orthologues ‘TadB’ (K12510), ‘TadC’ (K12511), ‘Flp’ (K02651), ‘RcpA’ (K02280), ‘TadZ’ (K02282), F-type ATPase α-subunit (K02111), F-type ATPase β-subunit (K02112) and ATP/ADP translocase ‘Tlc’ (K03301). Reference sequences were clustered at 70% identity according to cd-hit^104^ v4.8.1, and a reference alignment was generated using MAFFT^75^ v7.453 at default settings. Protein sequences from Omnitrophota assemblies were then aligned against the reference alignment. The resulting alignments were reduced using the gappyout function of trimAl^73^ v1.4.rev22. Phylogenetic trees were constructed using IQ-Tree^74^ v1.6.8, with model testing restricted to general protein models WAG, LG, JTT, JTTDCMUT and PMB. Node support was based on 1,000 SH-like aLRT (alrt) test and 1,000 ultrafast bootstrap^77^ rounds.

### Assessment of taxonomic affiliations of Omnitrophota genes

To determine sources of genes in each class of Omnitrophota and possible host or syntrophic relationships, taxonomic assignments of translated ORFs from each class were assessed using Kraken2 (ref. ^105^) v2.1.2 with default settings. Two different databases were used: (1) a custom database using NCBI’s RefSeq complete bacterial, archaeal, viral, fungal and protozoan genomes and (2) the 140 GB ‘maxikraken2’ database^106^.

### qSIP

qSIP was performed previously in diverse soils to probe relationships between mineral content and microbial activity^48,107^ but were repurposed here to determine whether soil Omnitrophota are highly active. There were two experiments (*n* = 3 per experiment group): (1) utilization of ^18^O H_2_O to assess intrinsic growth and response to carbon addition in the form of root exudates or leaf litter and (2) utilization of ^18^O H_2_O or ^13^C with carbon amendments of glucose and oxalic acid to soil. 16S rRNA gene amplicon reads from qSIP experiments were processed using Qiime v2020.8. Paired-end reads were quality filtered, joined and trimmed to 150 nt, and SVs were identified using Dada2 within Qiime2. SVs were then classified using both classifiers as previously described. Atom fraction excess (AFE) values were calculated using the R package qsip v0.1.0 (github.com/bramstone/qsip). Utilization of labelled substrate (^18^O or ^13^C) was considered for taxa where both tails of the 95% confidence interval of AFE values were greater than zero.

### Inclusion and ethics

This research included local researchers as full authors, when possible, to recognize both logistical and intellectual contributions. No potential or listed authors were discriminated against on the basis of gender, race, ethnicity or any other factors not related to scientific contributions.

### Reporting summary

Further information on research design is available in the Nature Portfolio Reporting Summary linked to this article.

## Supplementary information

Supplementary Information Supplementary Notes 1–3 and Figs. 1–16.

Reporting Summary

Supplementary Tables 1–11 Supplementary Tables 1–11.

## Data Availability

All assemblies used are available through INDSC and IMG accession numbers indicated in Supplementary Table 1. Raw reads for tandem filtration experiments were submitted to the Sequence Read Archive under BioProject PRJNA841252. Raw reads for qSIP experiments are available from the Sequence Read Archive under BioProjects PRJNA701328 and PRJNA846758. Data used by this project are available under the following DOIs: 10.46936/10.25585/60000685, 10.46936/10.25585/60007600, 10.46936/10.25585/60000876 and 10.46936/10.25585/60001034. Newick files for all phylogenetic trees shown or discussed in the manuscript are available via Figshare at 10.6084/m9.figshare.c.6010411. Source data are provided with this paper.

## Source data

Source Data Fig. 1 Compiled genome data.
Source Data Fig. 2 Cell size data and genome IDs.
Source Data Figs. 3–5 Tabulated genome annotation data.
Source Data Fig. 6 qSIP taxonomy, count, sources and AFE data.
Source Data Extended Data Fig. 1 Raw CheckM markerset statistics output.
Source Data Extended Data Fig. 3 Sample counts per empo_3 wherein Omnitrophota classes were observed.
Source Data Extended Data Fig. 4 Genome sizes of Omnitrophota and other phyla.
Source Data Extended Data Fig. 7 Tabulated ORF taxonomic affiliation as assigned by Kraken2.

## Code availability

Custom code is available via Figshare at 10.6084/m9.figshare.c.6010411. The scripts in this repository were used to: (1) parse IQ-tree model information into a model string, (2) perform phylogenetic placement on a GTDB-subtree and then re-graft the tree onto its original position, (3) calculate AAI quickly for hundreds of genomes and (4) identify ‘previously used’ taxa as described in the Online Methods.
