# Supplementary material for: Hyperactive nanobacteria with host-dependent traits pervade Omnitrophota
Source: Nat Microbiol. 2023 Mar 16;8(4):727–44. doi: 10.1038/s41564-022-01319-1 (PMC10066038; doi:10.1038/s41564-022-01319-1)
Supplement: Supplementary file 1 — Supplementary Notes 1–3 and Figs. 1–16. [file 41564_2022_1319_MOESM1_ESM.pdf]

nature microbiology

Analysis

https://doi.org/10.1038/s41564-022-01319-1

Hyperactive nanobacteria with host-dependent traits pervade Omnitrophota

In the format provided by the authors and unedited

Hyperactive nanobacteria with host-dependent traits pervade *Omnitrophota*

Cale O. Seymour, Marike Palmer, Eric D. Becraft, Ramunas Stepanauskas, Ariel D. Friel, Frederik Schulz, Tanja Woyke, Emiley Eloe-Fadrosh, Dengxun Lai, Jian-Yu Jiao, Zheng-Shuang Hua, Lan Liu, Zheng-Han Lian, Wen-Jun Li, Maria Chuvochina, Brianna K. Finley, Benjamin J. Koch, Egbert Schwartz, Paul Dijkstra, Duane P. Moser, Bruce A. Hungate, Brian P. Hedlund*

* Correspondence to: brian.hedlund@unlv.edu

Supplementary Notes **Page**

1 **Supplementary Note 1.** *Omnitrophota* are members of the PVC superphylum and Ratteibacteria are likely not. 2
2 **Supplementary Note 2.** The OLB16 / SURF_12 lineage is distinct from *Omnitrophota*. 2
3 **Supplementary Note 3.** Extended discussion of *Omitrophota* physiology. 3

Supplementary Figures

1 **Supplementary Figure 1.** CheckM marker set and heatmap. 7
2 **Supplementary Figure 2.** UBCG marker set and heatmap. 8
3 **Supplementary Figure 3.** Concatenated protein phylogenies including low-quality genomes. 9
4 **Supplementary Figure 4.** GTDB r202 reference phylogeny with class-level taxonomy. 10
5 **Supplementary Figure 5.** Summary of synonymy checking algorithm. 11
6 **Supplementary Figure 6.** Comparison of COG reduction as percent and richness. 12
7 **Supplementary Figure 7.** Completeness of amino acid biosynthesis in *Omnitrophota*. 13
8 **Supplementary Figure 8.** Completeness of carbon and energy metabolisms in *Omnitrophota*. 14
9 **Supplementary Figure 9.** Metabolic markers in *Omnitrophota* genomes. 15
10 **Supplementary Figure 10.** Expanded genome feature table. 16
11 **Supplementary Figure 11.** Hydrogenase annotations in *Omnitrophota* genomes. 17
12 **Supplementary Figure 12.** TadC phylogeny. 18
13 **Supplementary Figure 13.** F-type ATPase α-subunit phylogeny. 19
14 **Supplementary Figure 14.** All giant ORFs in *Omnitrophota* sorted by order. 20
15 **Supplementary Figure 15.** ATP/ADP translocase phylogeny. 21
16 **Supplementary Figure 16.** Family-level qSIP results. 22

References 23

Supplementary Notes

Supplementary Note 1: *Omnitrophota* are members of the PVC superphylum and *Ratteibacteria* are likely not

Single-copy marker genes conserved among the species representatives of *Omnitrophota* support the inclusion of *Omnitrophota* within the PVC superphylum. In addition to the previously observed[1] PVC-specific marker domain TIGR04137, *Omnitrophota* also possess the PVC-specific marker domain TIGR04138 (**Supplementary Table 11**). PVC bacteria tend to systematically lack otherwise highly conserved bacterial marker genes[2]. This holds true for the *Omnitrophota*. Except for one occurrence, *Omnitrophota* lack a homolog for the cell division-related gene *ftsZ* and ribosomal assembly protein *ftsY* and uniformly lack the L30 ribosomal protein. According to a blastp[3] search, the protein sequence for the anomalous *ftsZ* shared 89 percent identity over 100 percent query coverage with a protein from an uncultured archaeon (acc no. VVB79106.1), with other hits to members of the candidate phylum Pacearchaeota. The protein sequence for *ftsY* shared 50 percent identity with a protein from the candidate phylum Microarchaeota (acc no. HGG68819.1). The appearance of these proteins represents either bin contamination or very recent horizontal gene transfers from archaea to *Omnitrophota*. The absence of ribosomal protein L30 and *ftsZ* are common features within the PVC bacteria, but the lineage-wide absence of *ftsY* is seen only in the *Omnitrophota*. Based on these same markers, the *Ratteibacteria* likely represent a novel phylum. Two previous releases of the GTDB report *Ratteibacteria* as a class (UBA8648) within *Omnitrophota*. Members of the *Ratteibacteria* lack the PVC-specific marker genes TIGR04137 and TIGR04138 and possess genes encoding *ftsZ*. These characteristics exclude them from the *Omnitrophota* and the PVC superphylum. Moreover, a full-length bac120 marker set phylogeny places the *Ratteibacteria* in a supported clade outside of the PVC superphylum, adjacent to *Elusimicrobia* (**Figure 1**). *Ratteibacteria* do, albeit sparsely, encode the uncharacterized domain PF10915, which is otherwise only found in the genomes of species of *Chlamydiae*. These results indicate that, while the two phyla are relatively closely related, the *Ratteibacteria* comprise a novel lineage distinct from the *Omnitrophota*. Nevertheless, the proximity of these two lineages and the disjunction of PVC-specific marker genes between them presents the hypothesis that the root of the PVC superphylum may lie in between the *Ratteibacteria* and *Omnitrophota*.

Supplementary Note 2: The OLB16 / SURF_12 lineage is distinct from *Omnitrophota*

Historically, discussion of "Omnitrophica" has included singleton genome, OLB16[4]. The name for this genome in the NCBI database is currently "Omnitrophica bacterium"; when it was first analyzed, the OLB16 genome was thought to be the first high-quality genomic representation for the phylum. This conclusion was based on the adjacency of 16S rRNA gene sequences on a sparse phylogenetic tree. However, the conclusion was flawed, even at the time: the OLB16 16S rRNA gene did not form a supported, monophyletic grouping with the available "Omnitrophica" 16S rRNA gene sequences[4]. All versions of the GTDB report that OLB16 is a phylum distinct from the *Omnitrophota*[5]. The genomic marker trees generated and discussed here recapitulate this result: OLB16 does not form a monophyletic grouping with *Omnitrophota*. Moreover, the OLB16 genome encodes all of the markers that are otherwise absent from *Omnitrophota*: L30 ribosomal protein, ftsY, and ftsZ. These features indicate that, despite being ostensibly named an "Omnitrophica bacterium", the OLB16 genome does not originate from a member of the phylum *Omnitrophota*.

The conclusions of methanotrophy drawn from the SURF_12[6] genome apply to the OLB16 lineage—not *Omnitrophota*. Momper et al. (2017)[6] discovered a methane monoxogenase within the genome SURF_12. From this gene, the authors infer methane oxidation as a possible metabolism of SURF_12. Noted by the authors as well was a paraphyly of the OP3/Omnitrophica genomes that were included in their study. This paraphyly forms two separate clades: one clade includes SURF_12 and OLB16, while the other clade corresponds to the many of the same *Omnitrophota* genomes discussed here. Using the GTDB, a follow-up analysis of the SURF_12 genome[7] revised and affirmed its affiliation with the GTDB phylum OLB16. This resolves the paraphyly and maintains the conclusion of Momper et al. (2017)[6]—methanotrophy may indeed be a feature of the OLB16 lineage. No signal of methane oxidation was otherwise observed among the *Omnitrophota* as defined here. It is unlikely that this metabolism is present in the phylum.

Supplementary Note 3: Extended discussion of *Omnitrophota* physiology

Many catabolic and anabolic pathways were complete. Genes involved in the three-carbon module of glycolysis (M00002) are extremely common among members of *Omnitrophota*, with nearly every genome encoding a full pathway. Genes involved in the cleavage and interconversion of hexoses were less common, however, suggesting some conserved gaps in Embden-Meyerhoff glycolysis. Fructose-bisphosphate aldolase (K01623, K01624, K01625) (**Table S5**) shows some degree of variability between classes: these genes are common in *Gorgyraia* and 4484-213, rare in the *Omnitrophia* and *Velamenicoccia*, and absent in 2-02-FULL-51-18 and *Aquiviventia*. Glucose-6-phosphate isomerase (K01810, K06859, K13810, or K15916), catalyzing the interconversion between glucose-6-phosphate and fructose-6-phosphate in glycolysis, is absent from the 2-02-FULL-51-18 and uncommon among the *Aquiviventia*. Despite this, genes encoding the non-oxidative phase of the pentose phosphate pathway (M00007) are plentiful across the phylum, providing a possible alternative to glycolysis for the cleavage of hexoses where one or more of the necessary genes are absent. Numerous multiple-sugar ABC transporters (KEGG: K02025-27, K10112, K10117-19) are commonly encoded by members of the phylum. Across the phylum, however, the phosphotransferase system was missing or incomplete, suggesting that glucose utilization is uncommon in *Omnitrophota*. Together, these features suggest a conserved chemoheterotrophic metabolic potential by which non-glucose di- and oligosaccharides are used as carbon and energy sources. 4484-213 stands as a stark exception to this, however: the two genomes from this lineage lack multiple-sugar transporters entirely, but uniformly possess an array of amino acid transporters. Members of this class may, therefore, instead use a chemoheterotrophic metabolism centered around the interconversion and subsequent catabolism of amino acids. However, these conclusions will likely be revised pending the expansion of genomic representation for the 4484-213. The oxidative phase of the pentose phosphate pathway (M00006; **Supplementary Table 5**) and purine or pyrimidine degradation pathways (M00546, M0004, **Supplementary Table 5**) were absent or extremely rare across the phylum. The non-oxidative phase and subsequent steps in the biosynthesis of LPS and nucleotides were common across the phylum. This suggests that nucleotides are synthesized by members of *Omnitrophota* and not consumed and that nucleotides are not the preferred carbon source of any *Omnitrophota*.

Genes encoding energy conservation pathways were ubiquitous in *Omnitrophota* genomes, and each class followed either an acetogenic or respiratory scheme (**Figure 3**, **4; Extended Data Figure 5, 6**). Genomes of most species assigned to the classes *Gorgyraia* (32/39) and *Velamenicoccia* (73/113) encode the key genes for sugar transport, Embden-Meyerhof glycolysis,

ferredoxin reduction via pyruvate:ferredoxin oxidoreductase, and acetogenesis via phosphotransacetylase (Pta) and acetate kinase (Ack) (**Figure 3**, **4a**). This acetogenic pathway yields ATP from the oxidation of sugars via glycolysis and the ATP-yielding hydrolysis of acetyl-CoA to acetate via acetyl-P[8]. These genomes also encode a highly conserved Rnf complex. When used by acetogenic bacteria, Rnf complexes serve to restore NAD^+ and oxidized ferredoxin pools[9], or in reverse, to generate an electrochemical gradient capable of powering an ATPase[10]. PEP carboxykinase provides another possible source of ATP while simultaneously generating oxaloacetate as a connection between glycolysis (**Supplementary Table 4**) and a "horseshoe"-type TCA cycle (**Figure 4a**, **Supplementary Table 6**), as described by Williams et al.[11]. Fumarate hydratase and malate dehydrogenase are present and serve either in redox balance[11] or to recycle fumarate generated through arginine[11] or ADP biosynthesis. Additionally, although very few genes for sulfur metabolism were annotated by KEGG or Metabolic (**Supplementary Table 5, Supplementary Fig. 9**), 23 of these genomes encode one or more putative reversible desulfovirdin-type dissimilatory sulfite reductases (DsrA, COG2221), which could either reduce sulfite to sulfide via hydrogenases or oxidize sulfide to sulfur[12], as has been suggested for SKK-01 and supported by an abundance of intracellular sulfur globules[13,14]. Considering species representative genomes, acetogenesis was thus predicted in 96 species, mapping to seven of the ten *Gorgyraia* families and eight of the ten *Velamenicoccia* families (**Figure 3**, **Supplementary Table 5**). These patterns also held for lower completeness genomes (**Supplementary Fig. 10**); however, differences exist among these putative acetogens based on the variable presence and completeness of the Wood-Ljungdahl pathway (WLP) and the types of hydrogenases (**Supplementary Fig. 11**) and ATPases present.

The most basic acetogenic pathway enabling fermentation of sugars is present in genomes of 14 species in the *Gorgyraia* families *Gorgyraeaceae*, *Taenaricolaceae*, and JABMRG01 and the *Velamenicoccia* family 4484-1171, as exemplified by *Makaraimicrobium thalassicum* (**Extended Data Figure 5a**). In *M. thalassicum*, the oxidative and ATP-yielding reactions of the Embden-Meyerhof-Parnas pathway and acetogenesis could be coupled to redox balancing and cation pumping reactions of the Rnf complex and the biomass precursor-generating reactions of the "horseshoe"-type TCA cycle. Absent any electron transport chain complexes, the metabolism of these organisms is based on the fermentation of sugars, with acetate and possibly fumarate as likely products.

All other putative acetogens have this basic metabolism but also encode components of the bacterial WLP, which could enable another route for acetogenesis or for fixation of CO and, in some cases, CO_2. The methyl branch of the WLP was complete in these genomes (i.e., Fhs, FolD, MetF) except for formate dehydrogenase (K05299, K15022), suggesting formate[15] as a substrate for the methyl branch rather than CO_2 (**Figure 3, 4a; Supplementary Table 5**). The completeness of the carbonyl branch was variable. Twenty-four species in the *Velamenicoccia* families *Velamenicoccaceae*, *Profunditerraquicolaceae*, and DTHP01 and the *Gorgyraia* families FEN-1320 and CAIMPC01 have a truncated carbonyl branch lacking AcsA and its homolog CooS/F, as exemplified by *V. archaeovorus* (**Extended Data Figure 5b**). AcsA and CooS/F catalyze the conversion of CO_2 to CO; thus, in the absence of AcsA and CooS/F, the carbonyl branch could utilize CO for acetogenesis or biosynthesis via the carbon monoxide dehydrogenase AcsB-E

complex, but CO_2 cannot be fixed. As in *M. thalassicum*, *V. archaeovorus* could manage redox balance and energize the membrane via an Rnf complex; however, it also encodes a 4g nickel-iron membrane-bound hydrogenase and a cytoplasmic A3 iron-only hydrogenase (**Figure 3, 4a, Extended Data Figure 5b**). 4g hydrogenases have been proposed to couple the oxidation of H_2 or methylated organic compounds to ferredoxin reduction for CO_2 fixation[16], yet other group-4 hydrogenases reverse this process, coupling H_2 production to carbon monoxide or formate oxidation to CO_2[17]. Group A3 FeFe hydrogenases are electron bifurcating hydrogenases that are used for redox balance. The WLP enzymes can also catalyze the reverse reaction of acetogenesis, fixation of acetate to acetyl-CoA, and can serve to ligate coenzyme A to propionate or other short-chain fatty acids[18]. Direct utilization of propionate via acetate kinase (**Figure 4a**) and phosphate transacetylase is consistent with the enrichment of *Omnitrophota* in an anaerobic reactor community fed propionate at a high dilution rate[19]. All *Omnitrophota* 16S rRNA phylotypes in the reactor community mapped to the family *Profunditerraquicolaceae* within the *Velamenicoccia* (Genomes 318-418, **Figure 1**, **Supplementary Table 1**), which encode this pathway (**Supplementary Table 5**). Overall, the metabolic scheme of these organisms is an acetogenic pathway with the same core components as *M. thalassicum* for sugar utilization, but with additional capacity to conserve energy and incorporate additional carbon sources via a simplified WLP.

The other WLP-encoding *Gorgyraia* and *Velamenicoccaceae* species encode CooS/F and therefore potentially fix CO_2 in addition to CO. An example of this metabolism is *Fontincolimonas calida* (**Extended Data Figure 5c**), which also encodes a V-type ATPase and a 4g NiFe membrane-bound hydrogenase. This most complete acetogenic pathway is present in 19 species from the *Gorgyraia* families *Aquitaenarimondaceae*, JABMQH01, JABMRJ01, JABMSB01, 2-01-FULL-45-10, RBG-13-46-9, and UBA10183 and the *Velamenicoccia* families *Profunditerraquicolaceae*, *Ghiorseimicrobiaceae*, DTHP01, UBA12090, QZM4-1-127, and QZM4-1-77.

In contrast with the predominant metabolism of most *Gorgyraia* and *Velamenicoccia*, many species within the *Gorgyraia* order *Pluralincolimonadales* (e.g., *Pluralincolimonas frigidipaludosa*; **Figures 1, 3**) and *Velamenicoccia* order *Zapsychrales* (e.g., *Fredricksoniimonas* spp. **Figures 1, 3**) lack genes encoding acetate kinase and phosphate transacetylase and instead encode an acetyl-CoA synthetase along with diverse catabolic pathways. The four *Pluralincolimonadales* species all encode a reversible acetyl-CoA synthetase (TIGR02717) as well as a simplified electron transport chain including respiratory complex I (NADH dehydrogenase (PF00346)) and an F-type ATPase complex (M00157). Of the 39 species clusters in the *Zapsychrales*, 18 encode respiratory complex I, 13 encode respiratory complex II (succinate dehydrogenase (M00149)), and 37 encode an F-type ATPase complex. Only the species branching at the basal node of the order *Zapsychrales*, represented by Genome 314, encodes the WLP, suggesting loss of this pathway early in the evolution of the order. 13 species of *Zapsychrales* still encode a reversible acetyl-CoA synthetase (TIGR02717), suggesting acetogenesis or acetate utilization. The presence of cytochrome bd ubiquinol oxidase (M00153; 6 species) or cytochrome c oxidase (M00154; 1 species) indicates the genomic potential for aerobic respiration among some members of the order[20] (**Figure 3, Supplementary Table 5**). Genes encoding oxidoreductases

acting on particular terminal electron acceptors for anaerobic respiration such as nitrate, nitrite, and metals indicate a patchwork of respiratory systems in *Zapsychrales* and *Pluralincolimonadales* with little evidence of vertical inheritance.

Omnitrophia, *Aquiviventia*, and 2-02-FULL-51-18 lack the WLP altogether and instead encode diverse respiratory capabilities. Out of the 43 species clusters, genes encoding respiratory complex I are encoded by 34, yet genes encoding complex II are only encoded by 21 species. This complex is also rarely complete; genes encoding the membrane anchor component of complex II (K00242, K18859, or K18860) are missing from all *Omnitrophota* except members of *Aquiviventia* (**Supplementary Table 5**). Capacity for aerobic respiration via cytochrome bd ubiquinol oxidase (4 species) or cytochrome c oxidase (18 species) were also highly variable, although a large fraction of the *Aquiviventia* (10/13) appear to be aerobic via cytochrome c oxidase.

Alternative terminal oxidases in genomes of these three classes suggest a variety of options for terminal electron acceptors. Denitrification genes are encoded by some species of *Omnitrophia* (9/23), *Aquiviventia* (5/13), and class 2-02-FULL-51-18 (2/5), although none encode a complete denitrification pathway (**Figure 3**, **Supplementary Table 5**), suggesting syntrophic denitrification with other organisms. Genomes of some *Omnitrophia* (10/23) encode homologs of periplasmic cytochromes thought to be used by *Desulfovibrio ferrophilus* for dissimilatory metal reduction[21,22] (**Figure 3**). Some species clusters of *Omnitrophia* (12/23) and 2-02-FULL-18 (3/5) encode a putatively reversible acetyl-CoA synthetase (TIGR02717), suggesting acetogenesis or acetate utilization in addition to respiration. This gene is uncommon among species of *Aquiviventia* (1/13), however, indicating that respiration may be the major conserved energy metabolism of this lineage. Conductive pilins have been observed to facilitate direct electron transfer between syntrophic partners or to mineral surfaces[23]. Putatively conductive pilins[24] are predicted sparsely across species of *Omnitrophia* (11/23) as well as *Gorgyraia* (14/39) and *Velamenicoccia* (49/116) (**Figure 3**). However, known metal reduction pathways that would imply the use of these pilins for metal respiration are much less conserved, especially among species of *Gorgyraia* and *Velamenicoccia*.

Two specific examples of respiratory metabolisms are *Aquivivens invisus* in the *Aquiviventia* (**Extended Data Figure 6a**) and *Aquincolibacterium aerophilum* in the *Omnitrophia* (**Extended Data Figure 6b**), both predicted to be facultative anaerobes. *Aquivivens invisus* encodes transporters for sugars and amino acids and complete glycolysis and TCA cycle. Respiratory complexes I and II and a ubiquinone biosynthetic pathway are present, enabling electrons to flow into the quinone pool from either NADH, via complex I, or $FADH_2$ via complex II. Terminal electron acceptors could be oxygen, via cytochrome c oxidase, or nitrite. An F-type ATPase could couple a proton motive force to ATP synthesis. *Aquincolibacterium aerophilum* similarly encodes sugar and amino acid transport systems and Embden-Meyerhof glycolysis, but the TCA cycle lacks succinate dehydrogenase. Thus, it has a simplified respiratory system consisting of complex I, ubiquinone, and cytochrome bd ubiquinol oxidase, suggesting a microaerophilic lifestyle. A group A3 [FeFe] electron bifurcating hydrogenase and conductive pili could also serve to regenerate NAD^+ and FAD via hydrogen production or metal reduction, and an F-type ATPase could be used for chemiosmotic ATP synthesis.

Supplementary Figures

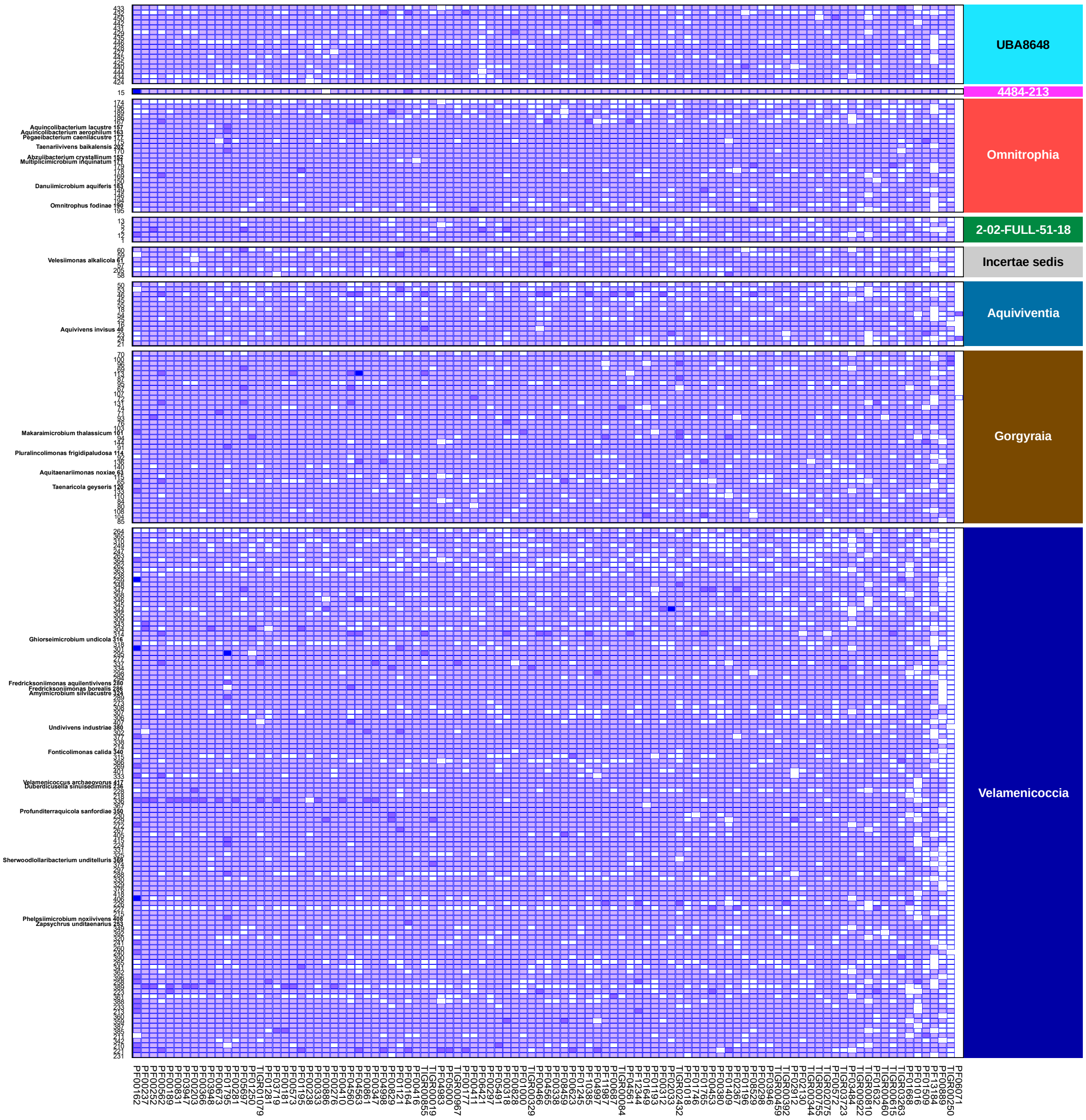

Supplementary Figure 1. CheckM General bacterial marker set and heatmap. Heatmap indicating the presence (purple) and absence (white) of markers included in the general bacterial marker set employed in CheckM for completeness and contamination estimated for Omnitrophota genomes. Markers present in multiple copies are indicated in darker shades. Numbered taxon labels refer to genome identifiers corresponding to **Table S1**, and proposed taxon names are indicated in bold. Genomes are grouped according to their class-level taxonomy.

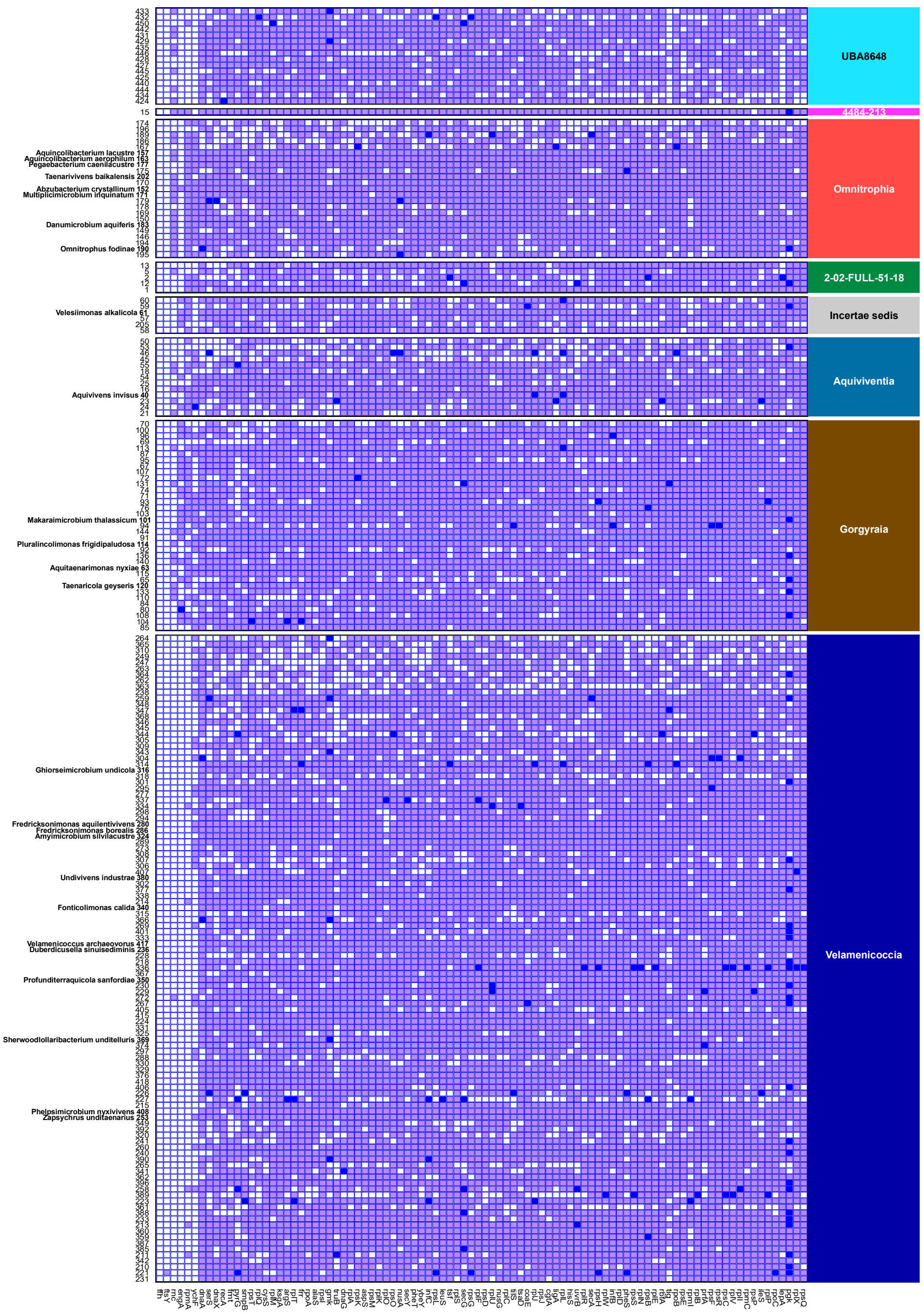

Supplementary Figure 2. UBCG marker set and heatmap. Heatmap indicating the presence (purple) and absence (white) of markers analyzed with the UBCG pipeline, in Omnitrophota and other closely related phyla. Markers present in multiple copies are indicated in darker shades. Numbered taxon labels refer to genome identifiers corresponding to **Table S1**, and proposed taxon names are indicated in bold. Genomes are grouped according to their class-level taxonomy.

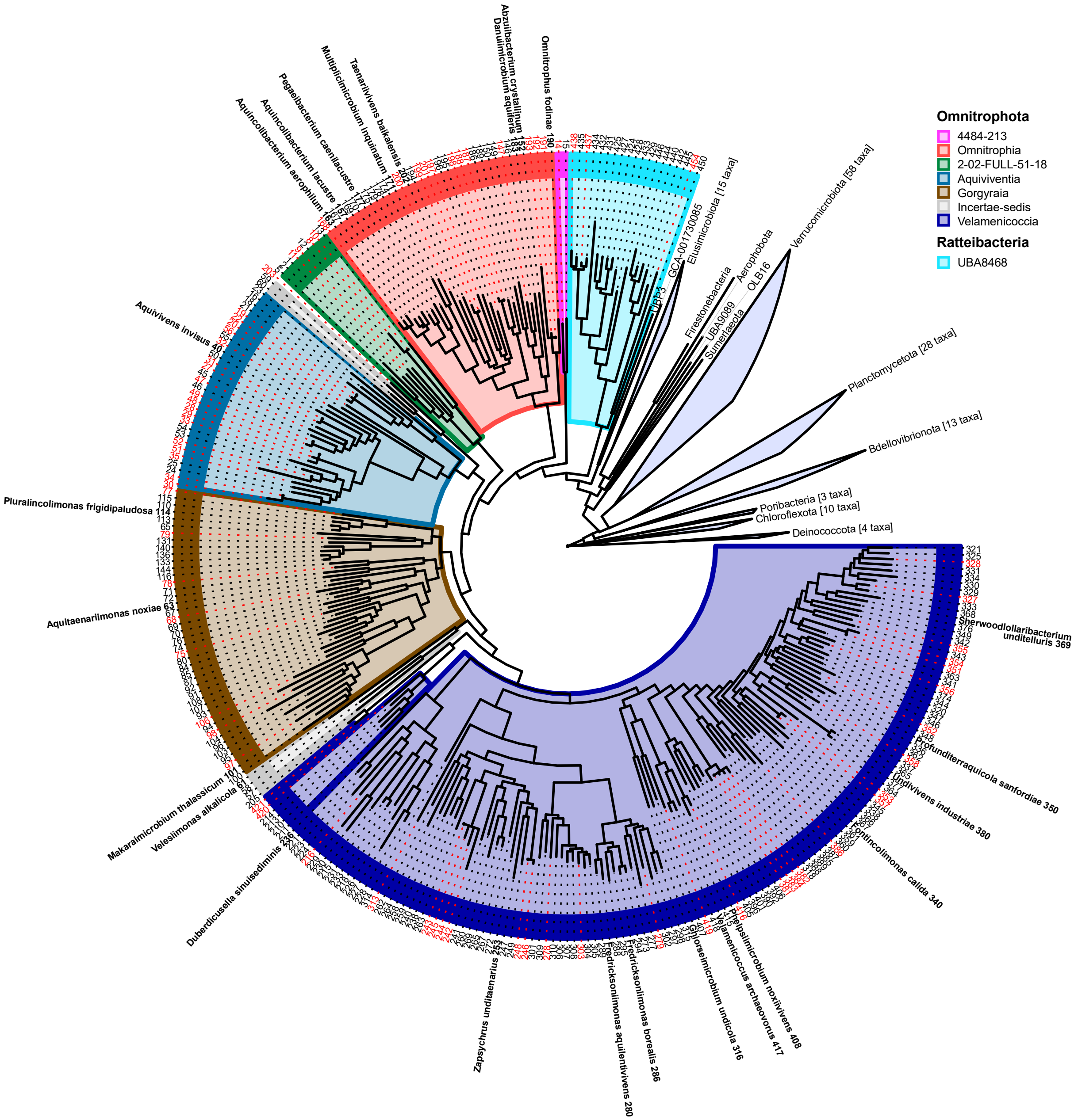

Supplementary Figure 3. Concatenated Bac120 phylogeny, including low-quality genomes. Bac120 marker set phylogenetic trees, including phylogenetically placed species, painted with Class - level taxonomy. Tips shown are genome identifiers and proposed taxon names in bold, corresponding to **Table S1**. Red tips indicate low-completeness, phylogenetically placed genomes.

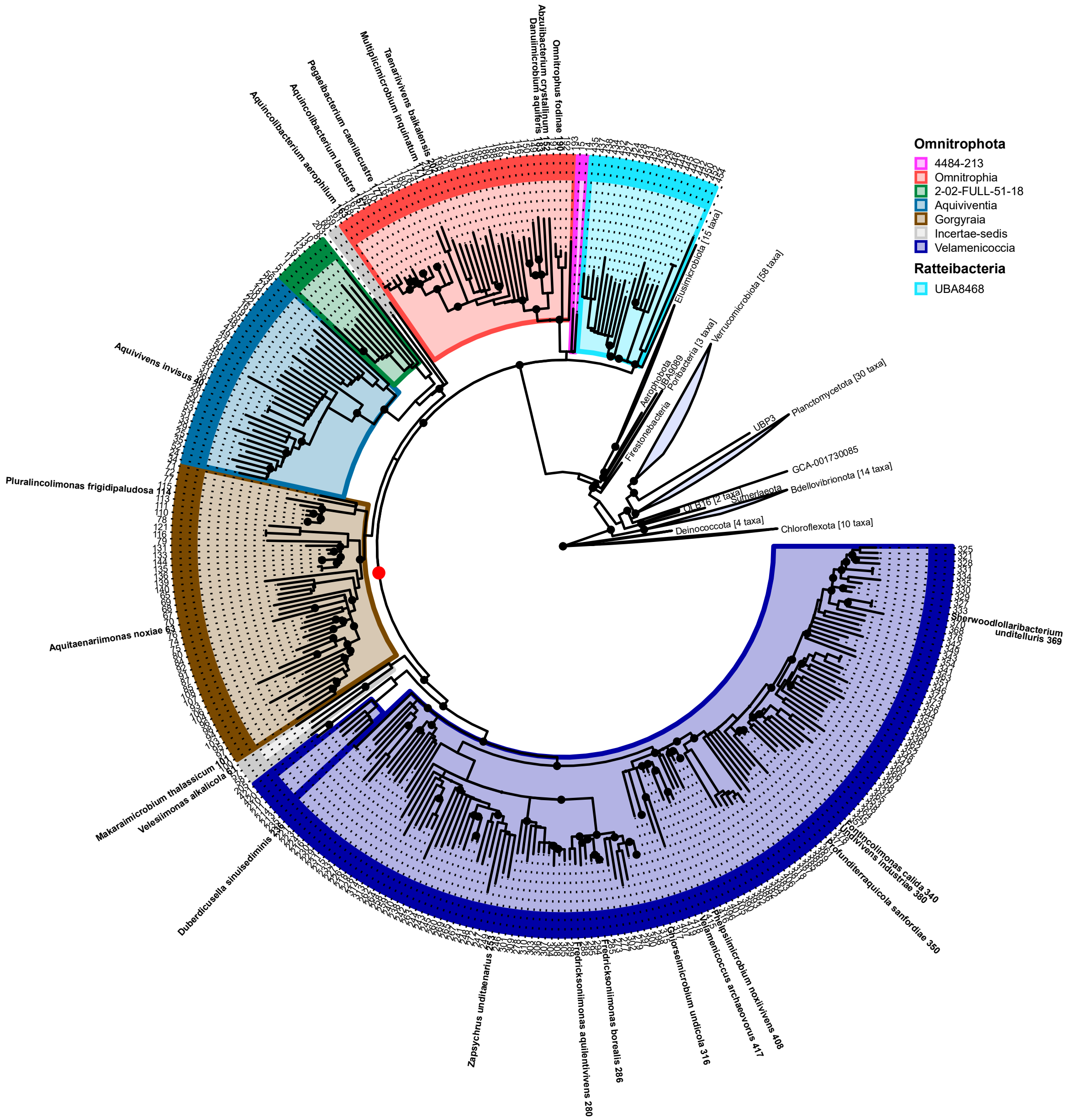

Supplementary Figure 4. GTDB r202 reference phylogeny with class-level taxonomy. Reference phylogeny used by r202 of the GTDB painted with the modified class-level taxonomy presented here. The node indicated in red is the parent node of the GTDB-class Koll11. Bold species are type material genomes.

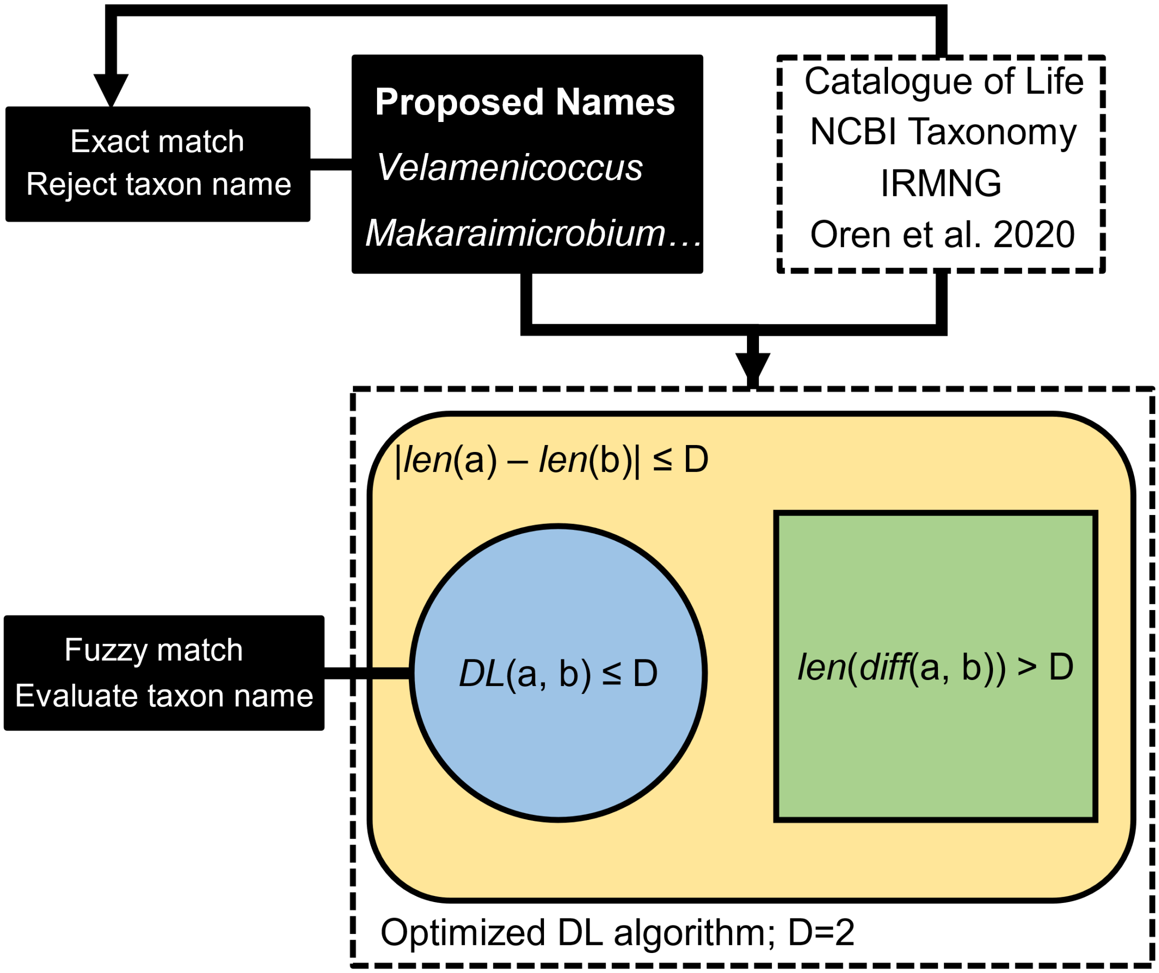

Supplementary Figure 5. Summary of synonymy-checking algorithm. Pictorial outline of the algorithm used to check the names of proposed taxa.

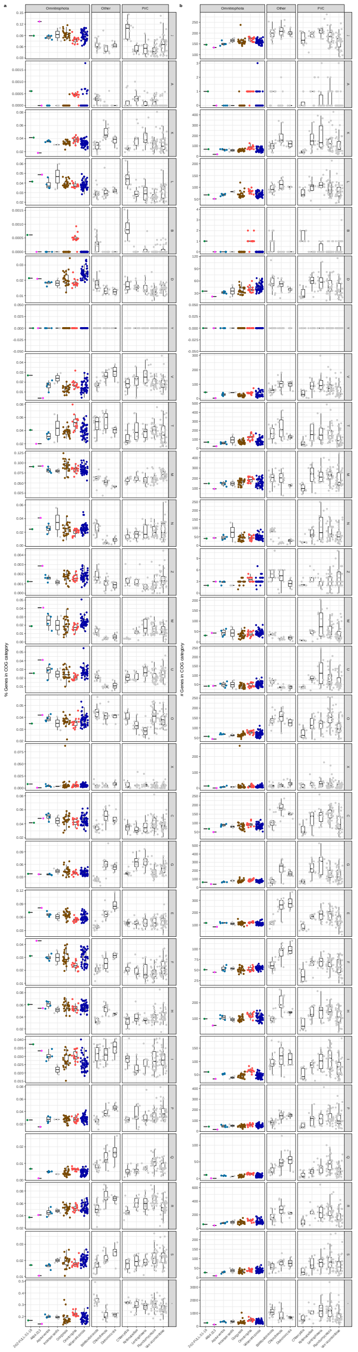

Supplementary Figure 6. Comparison of COG reduction as percent and richness. Comparison of NCBI COG enrichment based on (a) proportion and (b) count of ≥90% complete, ≥5% contaminated *Omnitrophota* and genomes from other bacteria. Members of other phyla are represented in gray. COG categories are arranged as facets. The y axis represents the proportion of genes encoded by a given genome that annotate to a COG within each category. The centermost divider of each boxplot represents the median. Upper and lower bounds of each box represent Q3 and Q2 respectively. Whiskers extend beyond Q3 and Q2 ±1.5 IQR. N = 204 genome assemblies (91 *Omnitrophota*, 13 *Bdellovibrionota*, 10 *Chloroflexota*, 4 *Deinococcota*, 28 *Planctomycetota*, and 58 *Verrucomicrobiota*.)

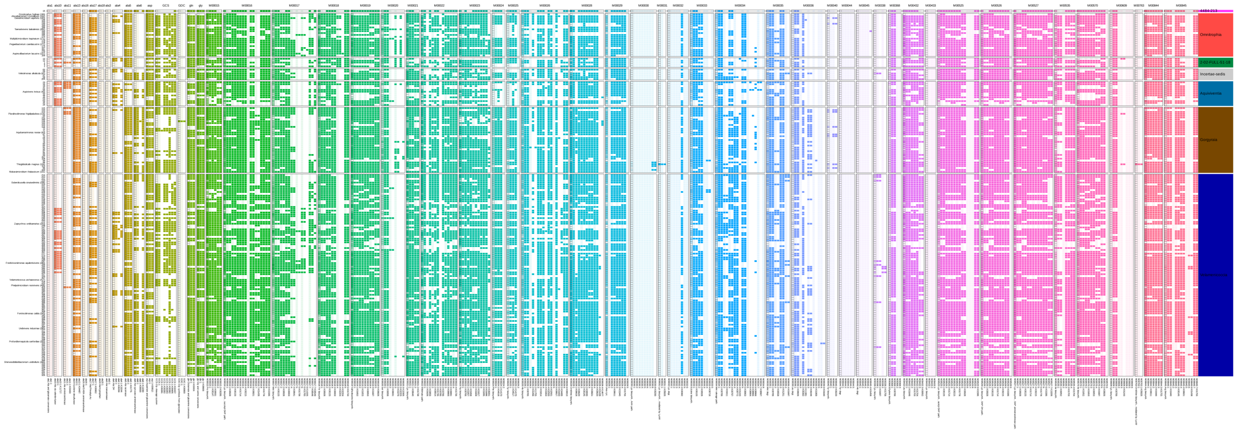

Supplementary Figure 7. Completeness of amino acid biosynthesis in *Omnitrophota*. Heatmap indicating the predicted completeness of amino acid metabolism KEGG modules in *Omnitrophota* genomes. Module columns are outlined in black. The color of a module tile indicates its completeness in a given genome: darker modules are more complete than lighter tiles. The individual KEGG genes involved in the module are shown in the columns to the right of each module column.

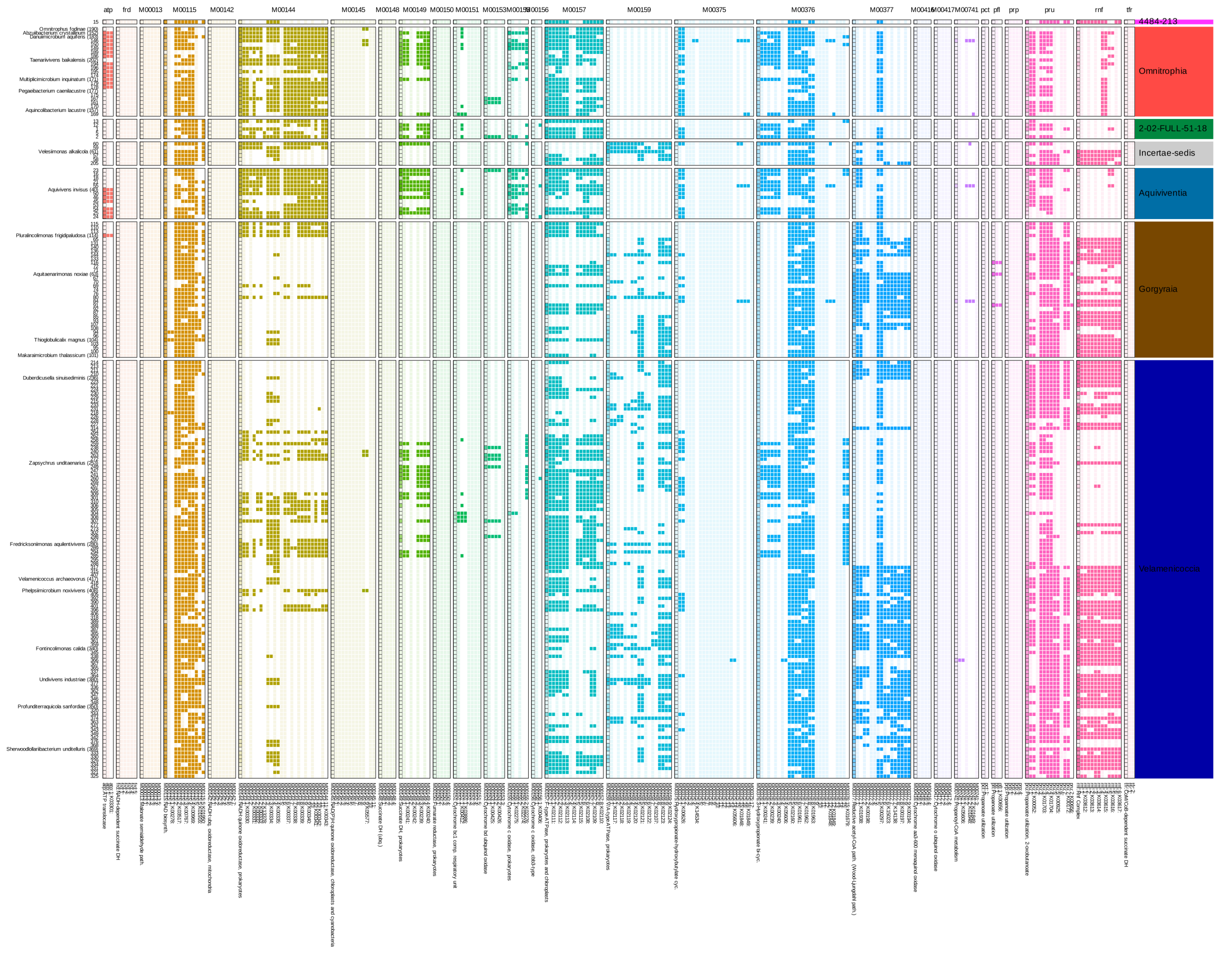

Supplementary Figure 8. Completeness of carbon and energy metabolisms in *Omnitrophota*. Heatmap indicating the predicted completeness of carbohydrate metabolism KEGG modules in *Omnitrophota* genomes. Module columns are outlined in black. The color of a module tile indicates its completeness in a given genome: darker modules are more complete than lighter tiles. The individual KEGG genes involved in the module are shown in the columns to the right of each module column.

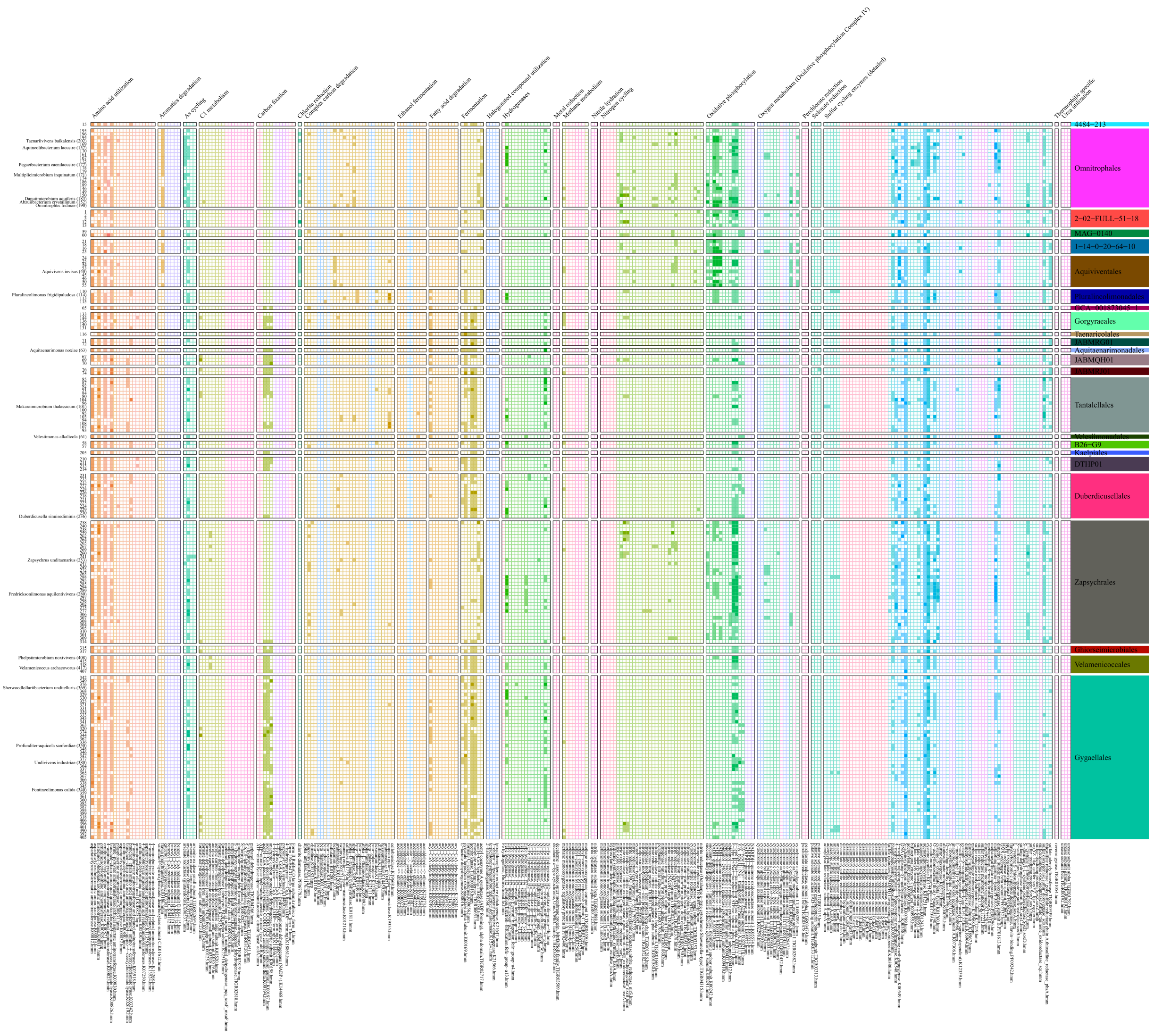

Supplementary Figure 9. Metabolic markers in *Omnitrophota* genomes. Heatmap of specific metabolism marker genes in *Omnitrophota* genomes. Some of the markers used were provided by the METABOLIC[25] software package. Darker colors indicate multiple copies of a given gene.

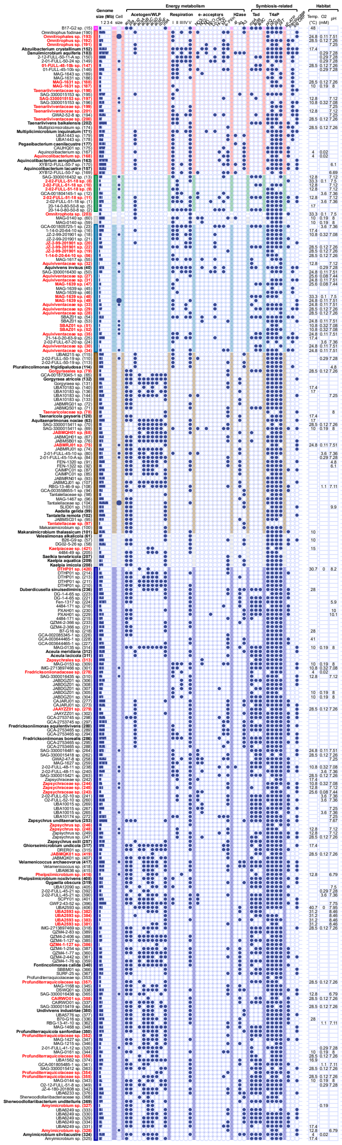

Supplementary Figure 10. Expanded genome feature table. Bars to the right of taxon names and in background reflect classes (**Figure 1**). Low-quality genomes are indicated in red. "Genome size" indicates the observed size of each genome. "Cell size" indicates evidence that the genome was sequenced from small cells (<0.5 microns, filled small circle) or large cells (>0.5 microns, filled large circle). "Acetogen/WLP" (Wood-Ljungdahl pathway and acetogenesis), "acs2" (acetyl-CoA synthetase), "acsABCDE" (CO dehydrogenase/acetyl-CoA synthase), "Respiration", "e- acceptors", and "H2ase" (hydrogenase) indicate genes predicted to encode proteins involved in energy metabolism. "Lo-O2" (cytochrome c oxidase complex); "Hi-O2" (cytochrome bd ubiquinol); "M+" (metal-reducing cytochromes); "e- Pilin" (conductive pili). Symbiosis-related genes include "T4aP" (type-4a pilus), "Tad" (tight adherence pilus), "sF-ATP ("symbiotic" type 2/3 F-type ATPase α-subunit), "Translocase" (ADP/ATP translocase) and "big ORF" (indicating the presence of a large ORF). "Temp", "O2", and "pH" indicate the observed temperature, oxygen concentration (mM), and pH of the sample from which each genome was sequenced. Numbers in parenthesis are unique genome identifiers as discussed in text.

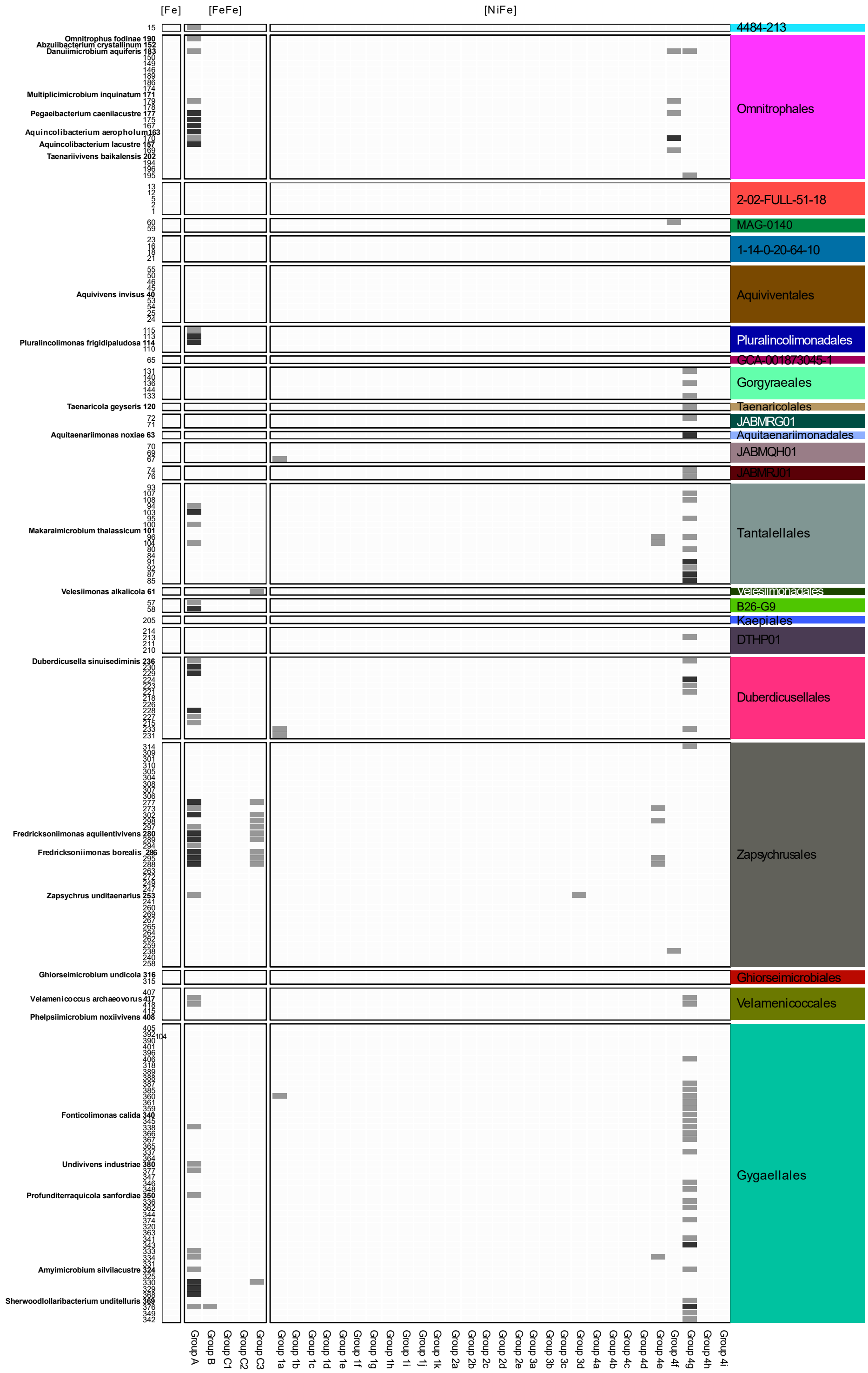

Supplementary Figure 11. Hydrogenase annotations in OP3 genomes. HydDB predictions for genes putatively encoding hydrogenase domains in *Omnitrophota* genomes. Darker colors indicate more domains per genome corresponding to each family of hydrogenase domains. Genome identifiers along with proposed taxonomic names in bold are indicated for each genome.

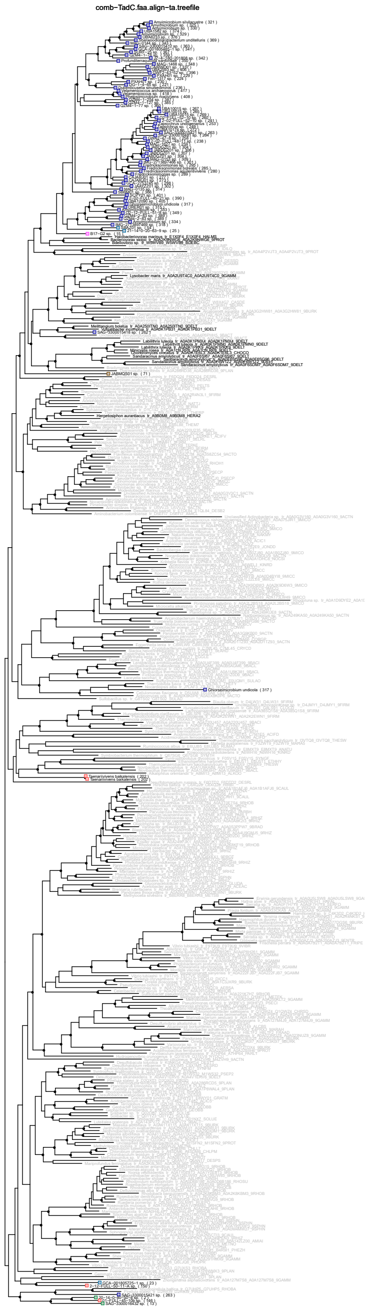

Supplementary Figure 12. TadC phylogeny. Phylogenetic tree constructed from the aligned protein sequences of TadC genes from *Omnitrophota* genomes and a selection of genes annotated as K12511 according to the Uniprot and KEGG databases. Genome identifiers along with proposed taxon names in bold correspond to **Table S1**. Supported nodes (SH-aLRT $\geq$ 80% and UFboot $\geq$ 95%) are indicated with a black dot. Colored points located at a tip indicate a sequence from an *Omnitrophota* genome, and the colors used correspond to those defined in **Supplementary Fig. 1**.

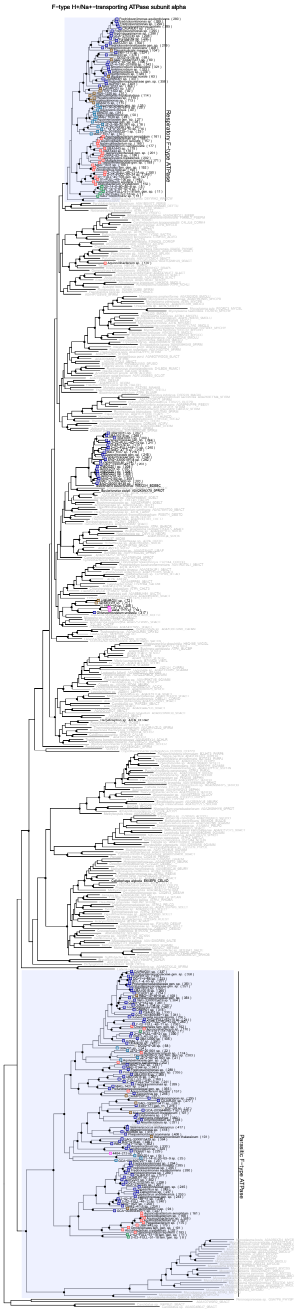

Supplementary Figure 13. F-type ATPase α subunit phylogeny. Phylogenetic tree constructed from the aligned protein sequences of putative F-type ATP-synthase subunit α genes from *Omnitrophota* genomes and a selection of genes annotated as K02111 according to the Uniprot and KEGG databases. Supported nodes (SH-aLRT ≥ 80% and UFboot ≥ 95%) are indicated with a black dot. Colored points located at a tip indicate a sequence from an *Omnitrophota* genome, and the colors used correspond to those defined in **Supplementary Fig. 1**.

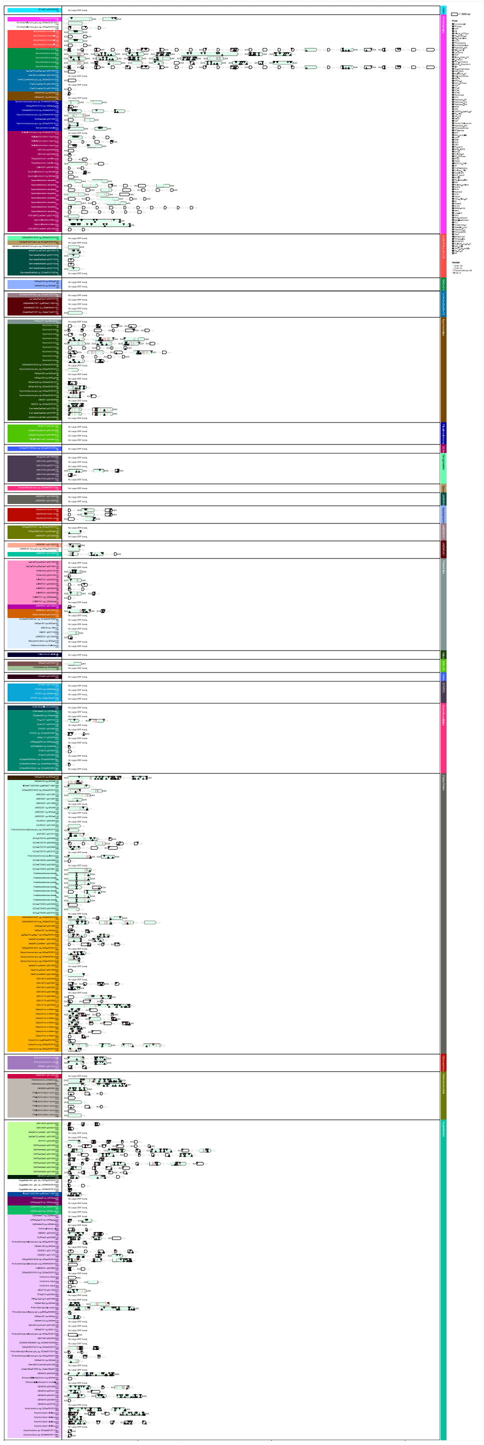

Supplementary Figure 14. All giant ORFs in *Omnitrophota* sorted by order. Diagram showing all giant ORFs from *Omnitrophota*, organized according to order. Genome identifiers along with proposed taxon names in bold correspond to **Table S1**. Pendants represent ORFs. Transmembrane sequence signals are displayed by the fill color of each pendant. Pendants without a transmembrane signal annotation are homologous to a large ORF from another genome.

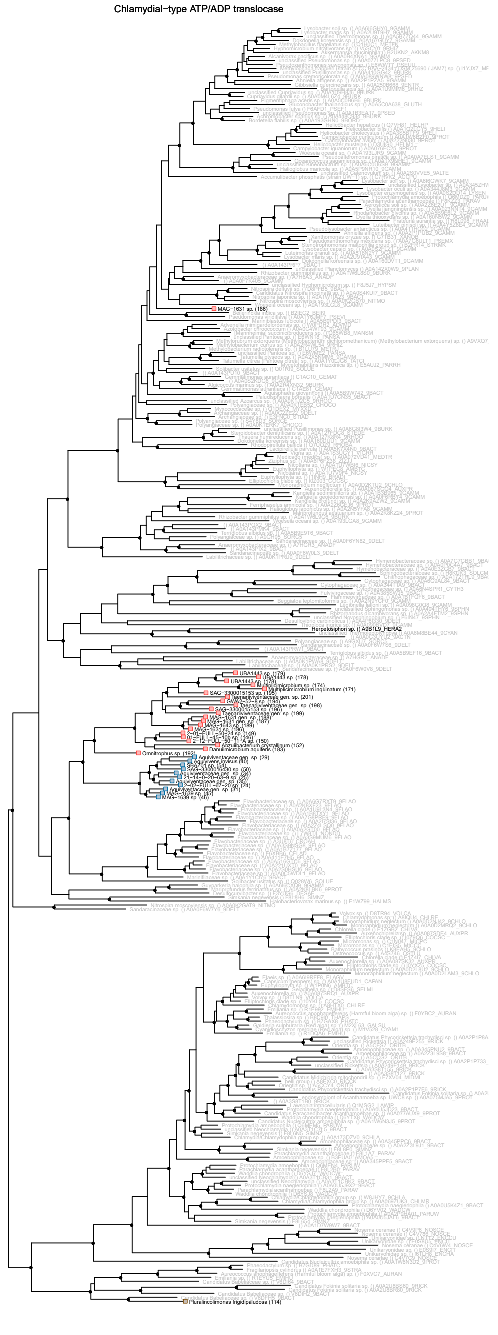

Supplementary Figure 15. ATP/ADP translocase phylogeny. Phylogenetic tree constructed from the aligned protein sequences of putative ADP/ATP translocase genes from *Omnitrophota* genomes and a selection of genes annotated as K03301 according to the Uniprot and KEGG databases. Genome identifiers along with proposed taxon names in bold correspond to **Table S1**. Supported nodes (SH-aLRT ≥ 80% and UFboot ≥ 95%) are indicated with a black dot. Colored points located at a tip indicate a sequence from an *Omnitrophota* genome, and the colors used correspond to those defined in **Supplementary Fig. 1**.

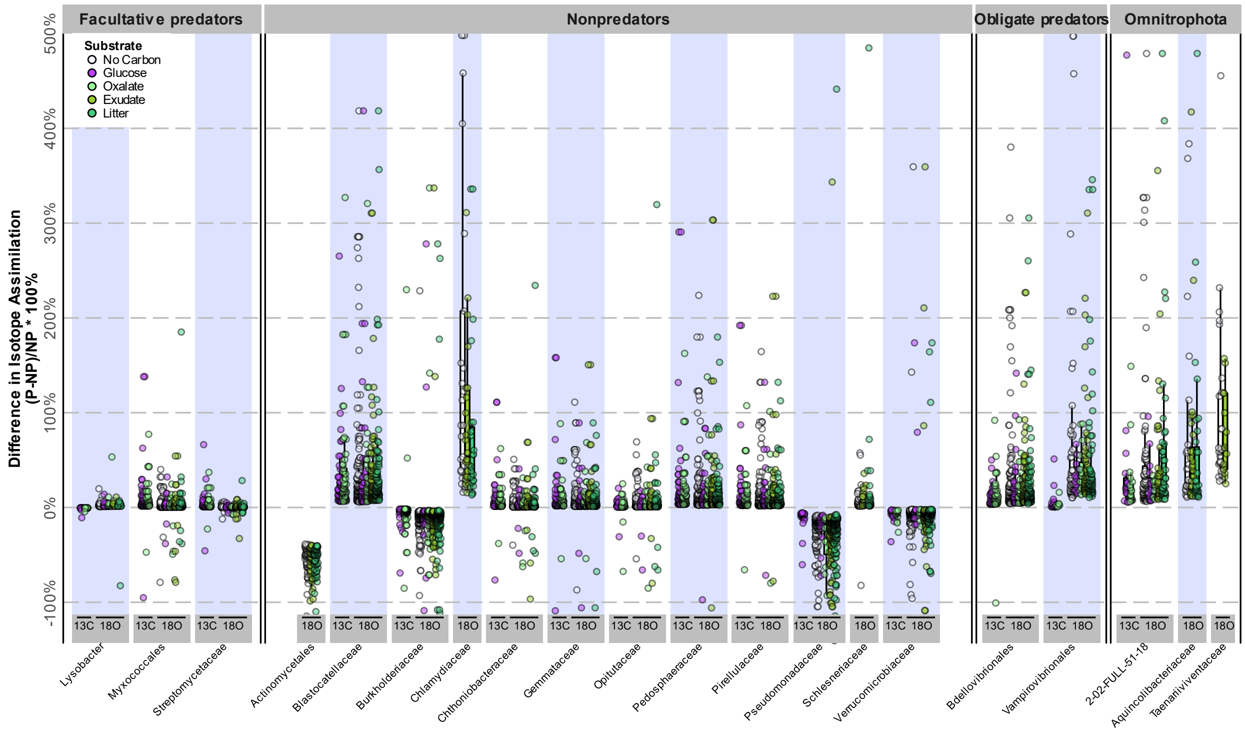

Supplementary Figure 16. Family-level quantitative stable isotope probing in diverse soils with control taxa included. Y-axis shows the percent difference between atom fraction excess (AFE) for a given taxon (P), compared to all non-predatory (NP) taxa from the same sample. Boxes display the median and inner quartiles while whiskers extend to the 95 percent confidence interval of the distribution of AFE ratios for a given taxon within each experimental group. N = 114 qSIP experiments.

References

1. Rinke, C. *et al.* Insights into the phylogeny and coding potential of microbial dark matter. *Nature* **499**, 431–437 (2013).
2. Lagkouvardos, I., Jehl, M.-A., Rattei, T. & Horn, M. Signature Protein of the PVC Superphylum. *Appl Environ Microbiol* **80**, 440–445 (2014).
3. Camacho, C. *et al.* BLAST+: architecture and applications. *BMC Bioinformatics* **10**, 421 (2009).
4. Speth, D. R., In ’t Zandt, M. H., Guerrero-Cruz, S., Dutilh, B. E. & Jetten, M. S. M. Genome-based microbial ecology of anammox granules in a full-scale wastewater treatment system. *Nat Commun* **7**, 11172 (2016).
5. Parks, D. H. *et al.* A standardized bacterial taxonomy based on genome phylogeny substantially revises the tree of life. *Nat Biotechnol* **36**, 996–1004 (2018).
6. Momper, L., Jungbluth, S. P., Lee, M. D. & Amend, J. P. Energy and carbon metabolisms in a deep terrestrial subsurface fluid microbial community. *ISME J* **11**, 2319–2333 (2017).
7. Momper, L., Casar, C. P. & Osburn, M. R. A metagenomic view of novel microbial and metabolic diversity found within the deep terrestrial biosphere. 2021.05.06.442964 Preprint at https://doi.org/10.1101/2021.05.06.442964 (2021).
8. Dittrich, C. R., Bennett, G. N. & San, K.-Y. Characterization of the Acetate-Producing Pathways in Escherichia coli. *Biotechnology Progress* **21**, 1062–1067 (2005).
9. Westphal, L., Wiechmann, A., Baker, J., Minton, N. P. & Müller, V. The Rnf Complex Is an Energy-Coupled Transhydrogenase Essential To Reversibly Link Cellular NADH and Ferredoxin Pools in the Acetogen Acetobacterium woodii. *Journal of Bacteriology* **200**, (2018).

10. Kuhns, M., Trifunović, D., Huber, H. & Müller, V. The Rnf complex is a Na + coupled respiratory enzyme in a fermenting bacterium, Thermotoga maritima. *Communications Biology* **3**, 1–10 (2020).
11. Williams, T. J., Allen, M. A., Berengut, J. F. & Cavicchioli, R. Shedding Light on Microbial "Dark Matter": Insights Into Novel Cloacimonadota and Omnitrophota From an Antarctic Lake. *Frontiers in Microbiology* **12**, 2947 (2021).
12. Steuber, J. & Kroneck, P. M. H. Desulfoviridin, the dissimilatory sulfite reductase from Desulfovibrio desulfuricans (Essex): new structural and functional aspects of the membranous enzyme. *Inorganica Chimica Acta* **275–276**, 52–57 (1998).
13. Kolinko, S. *et al.* Single-cell analysis reveals a novel uncultivated magnetotactic bacterium within the candidate division OP3. *Environmental Microbiology* **14**, 1709–1721 (2012).
14. Kolinko, S., Richter, M., Glöckner, F.-O., Brachmann, A. & Schüler, D. Single-cell genomics of uncultivated deep-branching magnetotactic bacteria reveals a conserved set of magnetosome genes. *Environmental Microbiology* **18**, 21–37 (2016).
15. Crowley, P. J., Gutierrez, J. A., Hillman, J. D. & Bleiweis, A. S. Genetic and physiologic analysis of a formyl-tetrahydrofolate synthetase mutant of Streptococcus mutans. *Journal of Bacteriology* **179**, 1563–1572 (1997).
16. Loh, H. Q., Hervé, V. & Brune, A. Metabolic Potential for Reductive Acetogenesis and a Novel Energy-Converting [NiFe] Hydrogenase in Bathyarchaeia From Termite Guts – A Genome-Centric Analysis. *Front. Microbiol.* **11**, (2021).
17. Greening, C. *et al.* Genomic and metagenomic surveys of hydrogenase distribution indicate H2 is a widely utilised energy source for microbial growth and survival. *ISME J* **10**, 761–777 (2016).

18. Hesslinger, C., Fairhurst, S. A. & Sawers, G. Novel keto acid formate-lyase and propionate kinase enzymes are components of an anaerobic pathway in Escherichia coli that degrades L-threonine to propionate. *Mol Microbiol* **27**, 477–492 (1998).
19. Tang, Y.-Q., Shigematsu, T., Morimura, S. & Kida, K. Effect of dilution rate on the microbial structure of a mesophilic butyrate-degrading methanogenic community during continuous cultivation. *Appl Microbiol Biotechnol* **75**, 451–465 (2007).
20. Baughn, A. D. & Malamy, M. H. The strict anaerobe Bacteroides fragilis grows in and benefits from nanomolar concentrations of oxygen. *Nature* **427**, 441–444 (2004).
21. Garber, A. I. *et al.* FeGenie: A Comprehensive Tool for the Identification of Iron Genes and Iron Gene Neighborhoods in Genome and Metagenome Assemblies. *Front. Microbiol.* **11**, (2020).
22. Deng, X., Dohmae, N., Nealson, K. H., Hashimoto, K. & Okamoto, A. Multi-heme cytochromes provide a pathway for survival in energy-limited environments. *Science Advances* **4**, eaao5682 (2018).
23. McGlynn, S. E., Chadwick, G. L., Kempes, C. P. & Orphan, V. J. Single cell activity reveals direct electron transfer in methanotrophic consortia. *Nature* **526**, 531–535 (2015).
24. Walker, D. J. *et al.* Electrically conductive pili from pilin genes of phylogenetically diverse microorganisms. *The ISME Journal* **12**, 48–58 (2018).
25. Zhou, Z., Tran, P., Liu, Y., Kieft, K. & Anantharaman, K. METABOLIC: A scalable high-throughput metabolic and biogeochemical functional trait profiler based on microbial genomes. *bioRxiv* 761643 (2019) doi:10.1101/761643.
