# Supplementary material for: Hyperactive nanobacteria with host-dependent traits pervade Omnitrophota
Source: Nat Microbiol. 2023 Mar 16;8(4):727–44. doi: 10.1038/s41564-022-01319-1 (PMC10066038; doi:10.1038/s41564-022-01319-1)
Supplement: Supplementary file 2 — Reporting Summary [file 41564_2022_1319_MOESM2_ESM.pdf]

nature portfolio

Corresponding author(s): Brian Hedlund

Last updated by author(s): Nov 17, 2022

Reporting Summary

Nature Portfolio wishes to improve the reproducibility of the work that we publish. This form provides structure for consistency and transparency in reporting. For further information on Nature Portfolio policies, see our Editorial Policies and the Editorial Policy Checklist.

Statistics

For all statistical analyses, confirm that the following items are present in the figure legend, table legend, main text, or Methods section.

n/a	Confirmed	
☐	☒	The exact sample size (*n*) for each experimental group/condition, given as a discrete number and unit of measurement
☐	☒	A statement on whether measurements were taken from distinct samples or whether the same sample was measured repeatedly
☐	☒	The statistical test(s) used AND whether they are one- or two-sided<br>*Only common tests should be described solely by name; describe more complex techniques in the Methods section.*
☒	☐	A description of all covariates tested
☐	☒	A description of any assumptions or corrections, such as tests of normality and adjustment for multiple comparisons
☐	☒	A full description of the statistical parameters including central tendency (e.g. means) or other basic estimates (e.g. regression coefficient) AND variation (e.g. standard deviation) or associated estimates of uncertainty (e.g. confidence intervals)
☐	☒	For null hypothesis testing, the test statistic (e.g. *F*, *t*, *r*) with confidence intervals, effect sizes, degrees of freedom and *P* value noted<br>*Give P values as exact values whenever suitable.*
☒	☐	For Bayesian analysis, information on the choice of priors and Markov chain Monte Carlo settings
☒	☐	For hierarchical and complex designs, identification of the appropriate level for tests and full reporting of outcomes
☒	☐	Estimates of effect sizes (e.g. Cohen's *d*, Pearson's *r*), indicating how they were calculated

Our web collection on statistics for biologists contains articles on many of the points above.

Software and code

Policy information about availability of computer code

Data collection	N/A
Data analysis	Custom code is via Figshare: https://doi.org/10.6084/m9.figshare.c.6010411. The scripts in this repository were used to: (i) parse IQ-tree model information into a model string, (ii) perform phylogenetic placement on a GTDB-subtree and then re-graft the tree onto its original position, (iii) calculate AAI quickly for hundreds of genomes, (iv) identify “previously-used” taxa as described in the Supplementary Text.

For manuscripts utilizing custom algorithms or software that are central to the research but not yet described in published literature, software must be made available to editors and reviewers. We strongly encourage code deposition in a community repository (e.g. GitHub). See the Nature Portfolio guidelines for submitting code & software for further information.

Data

Policy information about availability of data

All manuscripts must include a data availability statement. This statement should provide the following information, where applicable:

- Accession codes, unique identifiers, or web links for publicly available datasets
- A description of any restrictions on data availability
- For clinical datasets or third party data, please ensure that the statement adheres to our policy

All assemblies used are available through INDSC and IMG accession numbers indicated in Table S1. Raw reads for tandem filtration experiments were submitted to the Sequence Read Archive under BioProject PRJNA841252. Raw reads for qSIP experiments are available from the Sequence Read Archive under BioProjects

PRJNA701328 and PRJNA846758. Data from this project are available under the following DOIs: 10.46936/10.25585/60000685, 10.46936/10.25585/60007600, 10.46936/10.25585/60000876, 10.46936/10.25585/60001034. Newick files for all phylogenetic trees shown or discussed in the manuscript are available via Figshare: https://doi.org/10.6084/m9.figshare.c.6010411.

Human research participants

Policy information about studies involving human research participants and Sex and Gender in Research.

Reporting on sex and gender	N/A
Population characteristics	N/A
Recruitment	N/A
Ethics oversight	N/A

Note that full information on the approval of the study protocol must also be provided in the manuscript.

Field-specific reporting

Please select the one below that is the best fit for your research. If you are not sure, read the appropriate sections before making your selection.

☐ Life sciences ☐ Behavioural & social sciences ☒ Ecological, evolutionary & environmental sciences

For a reference copy of the document with all sections, see nature.com/documents/nr-reporting-summary-flat.pdf

Ecological, evolutionary & environmental sciences study design

All studies must disclose on these points even when the disclosure is negative.

Study description	This study involved analysis several types of data: (i) metagenomes and metagenome-assembled genomes; (ii) single-cell amplified genomes; (iii) cell size data estimated during fluorescence-activated cell sorting (FACS); (iv) cell size data estimated by differential filtration; (v) quantitative stable-isotope probing (qSIP)
Research sample	Most data were mined from NCBI or IMG. Sediment samples were collected for metagenomics and PCR assay from geothermal springs in Tenchong County, China and from the Spring Mountains in Nevada. Permits for all samples were obtained.
Sampling strategy	N/A
Data collection	Most data were obtained from NCBI. Sediment samples were collected by Hedlund and Friel from the Spring Mountains. Samples in China were collected by Liu, Lian, Jiao, and Hua
Timing and spatial scale	Precise sample dates are indicated in the manuscript.
Data exclusions	No data were excluded
Reproducibility	Locations and details for all sample collection and experiments are described in as much detail possible to promote reproducibility. For nomenclatural types, all raw data are available to allow reanalysis of sequence data.
Randomization	N/A
Blinding	N/A

Did the study involve field work? ☒ Yes ☐ No

Field work, collection and transport

Field conditions	N/A
Location	N/A
Access & import/export	N/A

Disturbance	N/A

Reporting for specific materials, systems and methods

We require information from authors about some types of materials, experimental systems and methods used in many studies. Here, indicate whether each material, system or method listed is relevant to your study. If you are not sure if a list item applies to your research, read the appropriate section before selecting a response.

Materials & experimental systems

n/a	Involved in the study
☒	☐ Antibodies
☒	☐ Eukaryotic cell lines
☒	☐ Palaeontology and archaeology
☒	☐ Animals and other organisms
☒	☐ Clinical data
☒	☐ Dual use research of concern

Methods

n/a	Involved in the study
☒	☐ ChIP-seq
☒	☐ Flow cytometry
☒	☐ MRI-based neuroimaging
